# USP11 regulates proliferation and apoptosis of human spermatogonial stem cells via HOXC5-mediated canonical WNT/β-catenin signaling pathway

**DOI:** 10.1007/s00018-024-05248-6

**Published:** 2024-05-09

**Authors:** Jun Gao, Zhipeng Xu, Weijie Song, Jiwei Huang, Wei Liu, Zuping He, Leye He

**Affiliations:** 1 Department of Urology, The Third Xiangya Hospital, Central South University, Changsha, China; 2 Key Laboratory of Model Animals and Stem Cell Biology in Hunan Province, School of Medicine, Engineering Research Center of Reproduction and Translational Medicine of Hunan Province, Hunan Normal University, Changsha, Hunan 410013 China; 3 Department of Pharmacy, The Third Xiangya Hospital, Central South University, Changsha, 410011 China

**Keywords:** USP11, Human spermatogonial stem cells, Proliferation and apoptosis, HOXC5, WNT/β-catenin pathway

## Abstract

Spermatogonial stem cells (SSCs) are capable of transmitting genetic information to the next generations and they are the initial cells for spermatogenesis. Nevertheless, it remains largely unknown about key genes and signaling pathways that regulate fate determinations of human SSCs and male infertility. In this study, we explored the expression, function, and mechanism of USP11 in controlling the proliferation and apoptosis of human SSCs as well as the association between its abnormality and azoospermia. We found that USP11 was predominantly expressed in human SSCs as shown by database analysis and immunohistochemistry. USP11 silencing led to decreases in proliferation and DNA synthesis and an enhancement in apoptosis of human SSCs. RNA-sequencing identified HOXC5 as a target of USP11 in human SSCs. Double immunofluorescence, Co-immunoprecipitation (Co-IP), and molecular docking demonstrated an interaction between USP11 and HOXC5 in human SSCs. HOXC5 knockdown suppressed the growth of human SSCs and increased apoptosis via the classical WNT/β-catenin pathway. In contrast, HOXC5 overexpression reversed the effect of proliferation and apoptosis induced by USP11 silencing. Significantly, lower levels of USP11 expression were observed in the testicular tissues of patients with spermatogenic disorders. Collectively, these results implicate that USP11 regulates the fate decisions of human SSCs through the HOXC5/WNT/β-catenin pathway. This study thus provides novel insights into understanding molecular mechanisms underlying human spermatogenesis and the etiology of azoospermia and it offers new targets for gene therapy of male infertility.

**Supplementary Information:**

The online version contains supplementary material available at 10.1007/s00018-024-05248-6.

## Introduction

Approximately 12% to 18% of couples at childbearing age are infertile, and half of the cases are attributed to male factors [1, 2]. Male infertility affects around 7% of the population [3]. Non-obstructive azoospermia (NOA) caused by testicular factors is one of the most challenging causes for male infertility [4, 5]. NOA are resulted from genetic factors, environmental factors, and endocrine disorders [4, 6], and it remains elusive about the etiology of NOA.

Spermatogenesis is a complex process which involves self-renewal and differentiation of spermatogonial stem cells (SSCs) [7], and it is regulated precisely by spatiotemporal gene expression and cell signaling [8, 9]. Any disruption at the three stages of spermatogenesis leads to occurrence of male infertility [10]. Studies on the mechanisms of SSCs are of great significance for reproductive medicine and regenerative medicine because they self-renew to maintain stem cell population, differentiate into spermatocytes and spermatids, and de-differentiate into pluripotential stem cells [11, 12]. SSCs reside within a microenvironment known as the niche, which is mainly composed of somatic cells and growth factors [13–15]. Within the niche, bioactive factors, e.g., CXCL12/CXCR4, FGF, and VEGFA act synergistically with GDNF to sustain SSC self-renewal [16–18]. On the contrary, retinoic acid (RA) induces SSC differentiation by activating BMP4 and downregulating GDNF expression [19, 20]. In recent years, with the development of stem cell transplantation technology, it might become feasible to treat male infertility through SSC transplantation [21, 22]. The number of SSCs is rare in human testicular tissues, while it has not yet been achieved for expansion and long-term culture of human SSCs [12, 23]. Moreover, regulatory molecules and signaling pathways of rodent SSCs are not applicable to humans, due to the distinct differences in cell types and phenotypes between human and rodent SSCs [23–25]. Therefore, it is of unusual significance to unveil regulatory networks by key genes and signaling pathways in mediating the fate decisions of human SSCs. We have established a human SSC line with biochemical phenotypes and functionalities similar to those of human primary SSCs [26], and thus this cell line could provide sufficient cells for us to explore molecular mechanisms of human SSC fate determinations.

Single-cell sequencing of human testicular tissues has provided a new avenue for in-depth gene analysis of testicular development, overcoming the limitation posed by the scarcity of human testicular samples available for SSC studies [13, 15]. We conducted a comprehensive analysis of three single-cell sequencing databases from human testicular tissues, including GSE109037, GSE112013, and GSE134144 [27–29]. Our analysis uncovered a differentially expressed gene, namely USP11, between human SSCs and other male germ cells across these databases. We revealed that USP11 was predominantly present in human SSCs by immunohistochemistry. Silencing and overexpression of USP11 significantly altered cell proliferation and apoptosis of human SSCs in vitro. Utilizing RNA-sequencing, double immunofluorescence, Co-immunoprecipitation (Co-IP), and molecular docking, we identified HOXC5 as a target of USP11. HOXC5 silencing suppressed proliferation and enhanced apoptosis of human SSCs, whereas overexpression of HOXC5 reversed the changes in cell proliferation and apoptosis of human SSCs induced by USP11 knockdown. HOXC5 silencing affected the expression levels of key molecules in the classical WNT/β-catenin pathway. Additionally, we observed a lower level of USP11 expression in NOA patients with spermatogenesis failure. As such, this study highlights a new genetic mechanism by which USP11 regulates the fate decisions of human SSCs and the pathogenesis of NOA and it provides new biomarkers for gene targeting male infertility.

## Results

### USP11 is predominantly expressed in human spermatogonia as analyzed by single-cell sequencing datasets

**Fig. 1:**
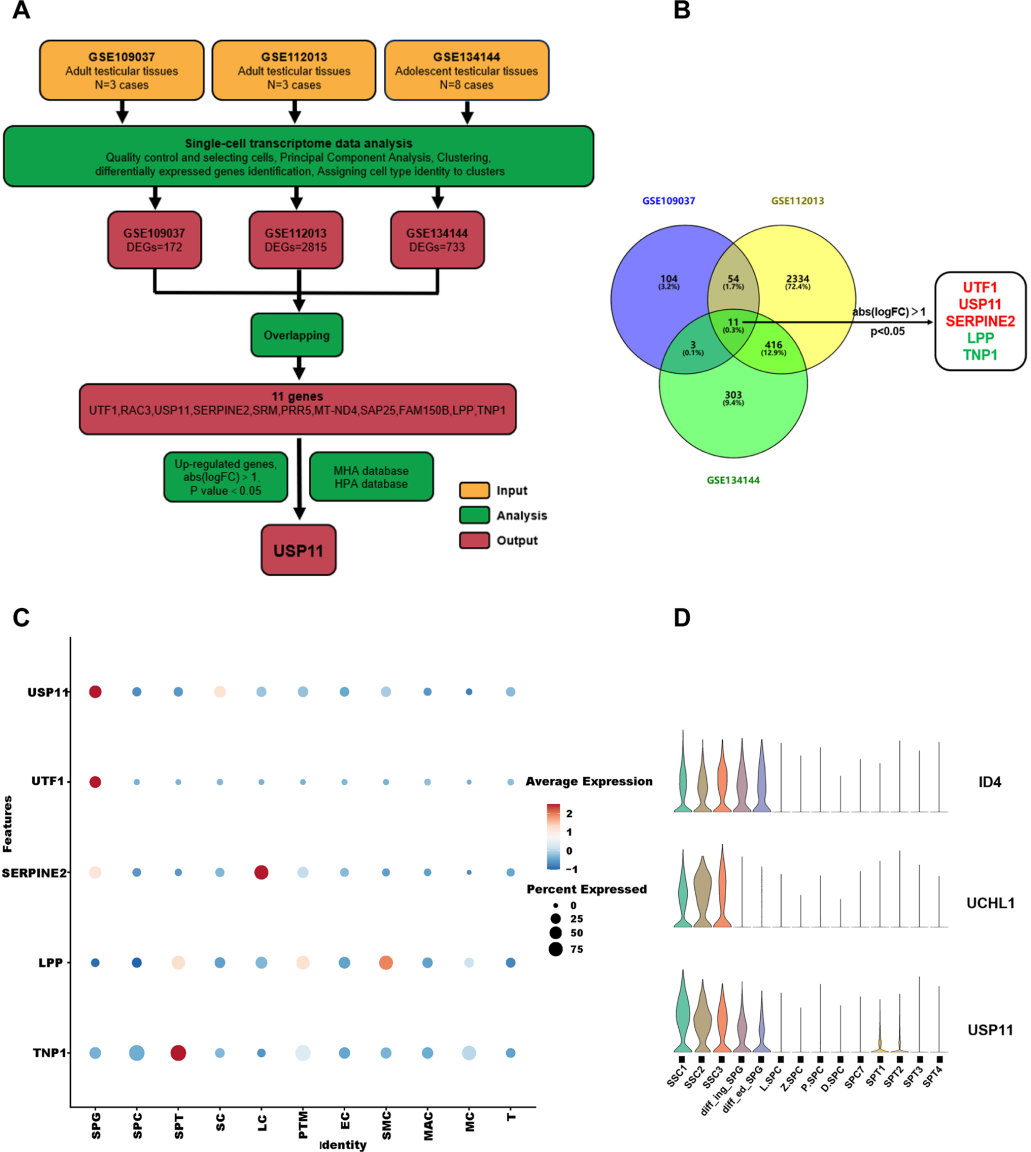
Identification of differentially expressed genes (DEGs) in SSCs via a comprehensive analysis of human testicular single-cell datasets. **A** Analysis pipeline for identifying genes with differential expression related to fate regulation of human SSCs. **B** Venn diagram of all DEGs in human SSCs from datasets GSE109037, GSE112013, and GSE134144. The genes highlighted in red represented the up-regulated DEGs, whereas those genes in green indicated the down-regulated DEGs. **C** Bubble diagrams revealed expression patterns of overlapping DEGs in all testicular cells. Average expression to map colors and percent expressed were used to map point sizes. **D** Violin plot showed the expression pattern of USP11, ID4, and UCHL1 in male germ cell lineages. Notes: *SPG*, spermatogonia; *SPC*, spermatocytes; *SPT*, spermatids; *SC*, Sertoli cells; *LC*, leydig cells; *PTM*, peritubular myoid cells; *EC*, endothelial cells; *SMC*, smooth muscle cells; *MAC*, macrophages; *MC*, mast cells; *T*, T cells

To identify specific genes that regulate the fate decisions of human SSCs, we analyzed publicly available single-cell sequencing datasets, including GSE109037, GSE112013, and GSE134144 (Fig. 1A). After quality control, selecting cells and clustering, we employed the standard Seurat package to classify all cells into 14 clusters, respectively. Cell type identities were assigned to clusters according to specific markers, e.g., ID4 and UCHL1 for human SSCs, FGFR3, KIT, and STRA8 for undifferentiated and differentiating spermatogonia, SYCP1, respectively, SPO11, OVOL2 and NME8 for spermatocytes, ZPBP, TNP2 and PRM2 for postmeiotic spermatids, WT1 and SOX9 for Sertoli cells, and IGF1 and CFD for leydig cells (Fig.S2A–C), and visualized by UMAP (Fig.S2D–F). Differentially expressed genes (DEGs) were selected between the SSC cluster and other cell clusters, and we obtained three DEG datasets. By overlapping these three gene datasets and applying filters (|log_2_ fold change| > 1, *p* < 0.05), we screened out five genes, with only three of them being up-regulated in SSCs (Fig. 1B). Ultimately, USP11 was chosen in this study for two key reasons. Firstly, the HMA database (http://malehealthatlas.cn/) shows that USP11 mRNA shares similarities in cellular distribution with hallmarks of human SSCs, e.g., UCHL1 and ID4, and USP11 expression is gradually decreased as male germ cells develop (Fig. 1C and D). Secondly, the HPA database (https://www.proteinatlas.org) indicates that USP11 is highly expressed in the human testis and it is predominantly present in human spermatogonia within seminiferous tubule (Fig. S3). All these results suggest that USP11 could be involved in controlling the destiny of human SSCs.

### USP11 is dominantly present in SSCs of human testes

**Fig. 2:**
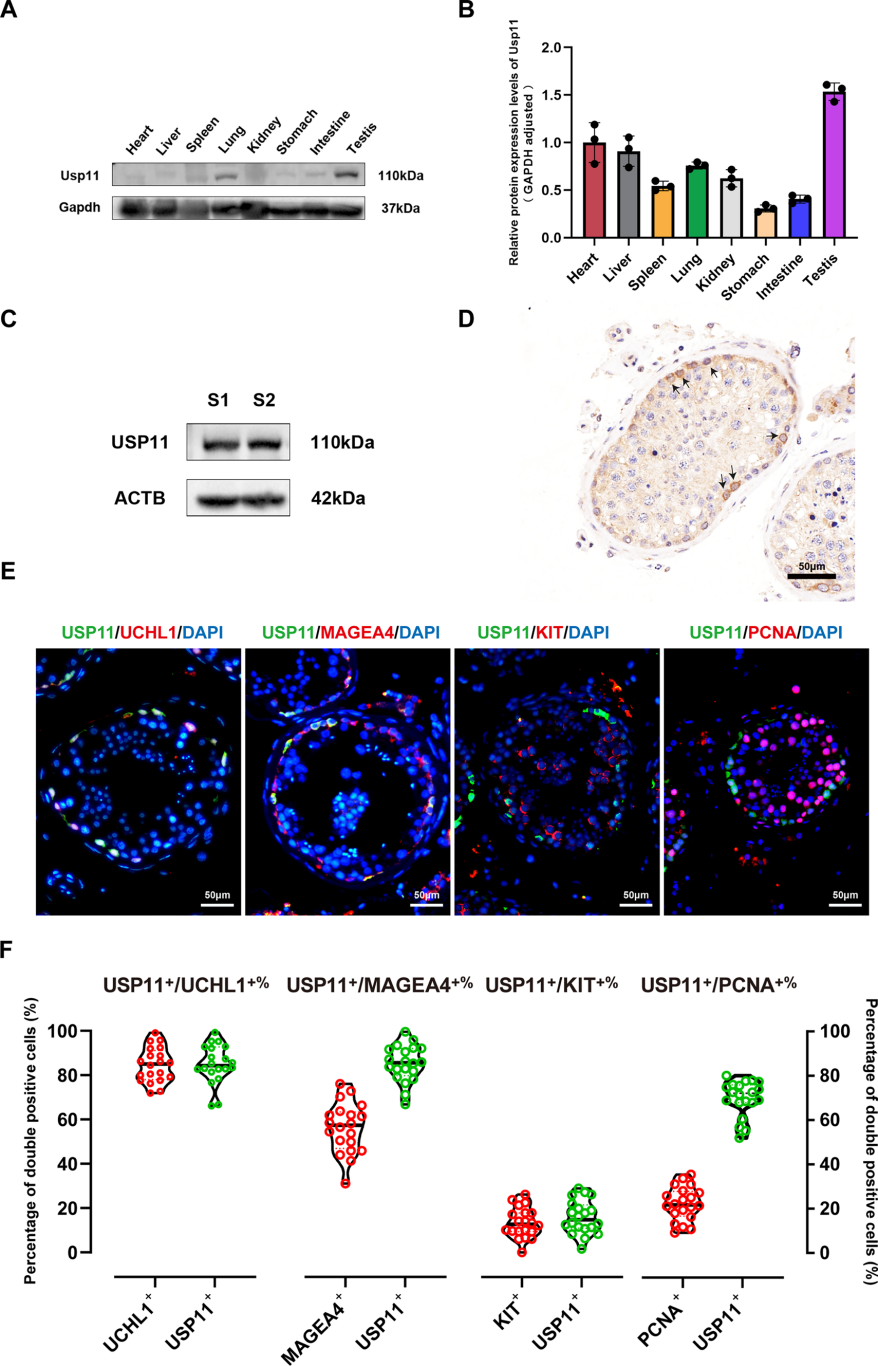
Expression of USP11 in normal human and mouse testicular tissues. **A**, **B**. Protein expression levels of USP11 were determined by Western blotting in various tissues of mice. **C** Western blotting detected the expression of USP11 in the human testicular tissues of two patients with normal spermatogenesis. **D** Immunohistochemistry illustrated cellular location of USP11 in human testicular tissues. Arrows indicated human spermatogonia that were positive for USP11. Scale bar: 50 µm. **E** Double immunostaining revealed co-expression of USP11 and UCHL1, MAGEA4, KIT, and PCNA in human testicular tissues. Green fluorescence represented USP11-positive cells, while red fluorescence denoted the expression of UCHL1, MAGEA4, KIT, and PCNA, respectively. Scale bars: 50 µm. **F** Violin plot of USP11 co-expression abundance with UCHL1, MAGEA4, KIT, and PCNA. Data were counted from at least 20 human seminiferous tubules

To determine tissue expression patterns of USP11, Western blots were conducted at various tissues of normal mice, and Usp11 was found to be expressed at a higher level in testicular tissue compared to other tissues of mice (Fig. 2A and B). Additionally, we observed that USP11 was expressed in human testicular tissues (Fig. 2C). Our immunohistochemistry (IHC) illustrated that USP11 was dominantly localized at human spermatogonia along the basement membrane of seminiferous tubules (Fig. 2D). To further reveal the subcellular distribution of USP11 in human testicular tissues, we performed double immunostaining  using several specific markers for human SSCs and spermatogonia. We found that USP11 was expressed in both the nuclei and cytoplasm of human SSCs, and USP11 was co-expressed with UCHL1 (Fig. 2E, first panel), a hallmark for human SSCs, and MAGEA4 (Fig. 2E, second panel), a marker for human spermatogonia. Twenty human seminiferous tubules were randomly selected in order to quantify USP11-positive cells. UCHL1 and MAGEA4 were expressed by approximately 85% of USP11-positive cells (84.92% ± 8.7% and 85.14% ± 8.23%, respectively) (Fig. 2F), and only 15.9% ± 7.54% of USP11-positive cells were co-localized KIT (Fig. 2E, third panel, Fig. 2F), a differentiating spermatogonial marker. Notably, 69.8% ± 8.02% of USP11-positive cells were co-expressed with PCNA (Fig. 2E, fourth panel, Fig. 2F), a cell proliferation-specific hallmark. Collectively, these results reflect that USP11 is dominantly expressed in SSCs of human testes.

### USP11 silencing leads to decreases in cell proliferation and DNA synthesis and an enhancement of apoptosis in human SSCs and influences cell cycle progression

To investigate the biological function of USP11 in human SSCs, we designed three sets of siRNAs to target *USP11*. The transfection efficiency of USP11 siRNAs was more than 85% as illustrated by Cy3-labelled siRNAs in human SSC line (Fig. S4A). Our qPCR and Western blots showed that mRNA and protein levels of USP11 were significantly decreased by USP11 siRNAs in human SSCs (Fig. S4B–D). Moreover, we established a human SSC line stably overexpressing USP11 (Fig. S5A–D).

**Fig. 3:**
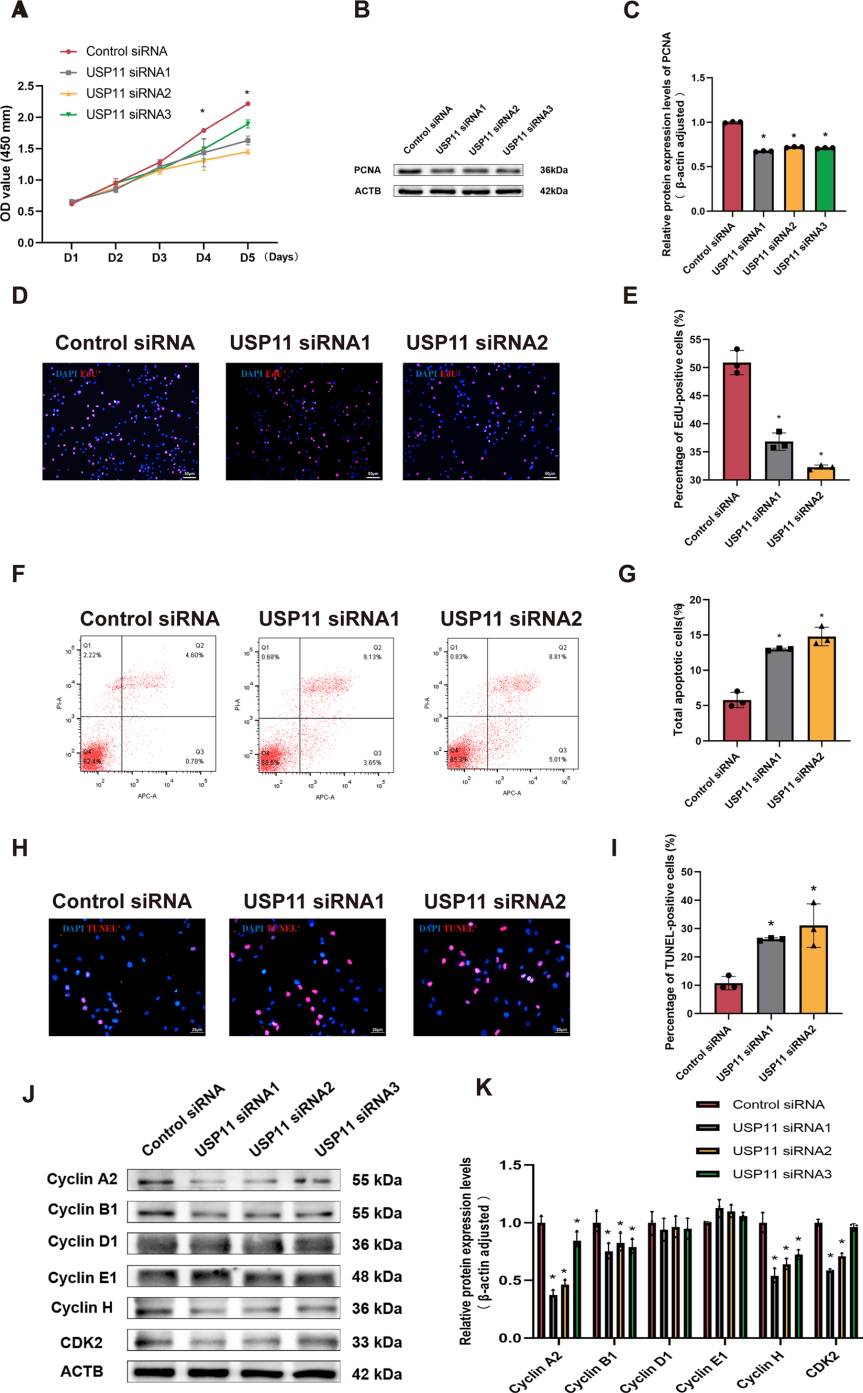
Effect of USP11 silencing or overexpression on proliferation  and apoptosis of SSCs. **A** Growth curves of human SSCs following five days of transfection of USP11 siRNA1-3 by CCK-8 assay. **B**, **C** Expression levels of PCNA was detected by Western blots in human SSCs after USP11 knockdown. **D**, **E** EdU assay indicated DNA synthesis in human SSCs following USP11 Knockdown. Red-fluorescence cells represented EdU-positive cells. Scale bars: 50 µm. **F**, **G** Annexin V-APC /propidium iodide (PI) staining and flow cytometry was utilized to measure apoptosis of human SSCs after USP11 silencing. **H**, **I** TUNEL assay was utilized to assess nuclear DNA fragmentation in human SSCs treatment with USP11siRNA1-2. Red-fluorescence cells showed TUNEL-positive cells. Scale bars: 25 µm. **J**, **K** Expression levels of cell cycle-related protein were measured in human SSCs treatment with USP11siRNA1-3 by Western blotting. “*” denoted a statistically significant difference with a *p*-value < 0.05

Cell Counting Kit-8 (CCK-8) assay was used to assess the proliferation ability of human SSCs after USP11 siRNA transfection for 5 days. Compared to control siRNA, the number of human SSCs was reduced significantly by USP11 siRNA1-3 at day 4 (Fig. 3A), whereas their number was significantly enhanced by overexpression of USP11 (Fig. S5F). Western blots showed that the level of PCNA of human SSCs was significantly decreased by USP11 siRNA1-3 compared with control siRNA (Fig. 3B and C), whereas their PCNA level was significantly enhanced by USP11 overexpression (Fig. S5C and E). After 48 h of cell transfection, we employed the 5-Ethynyl-2′-deoxyuridine (EdU) incorporation assay to assess DNA synthesis. EdU-positive cells were significantly diminished by USP11 siRNA1-2 in comparison to control siRNA (Fig. 3D and 3E) and increased by overexpression of USP11 in human SSCs (Fig. S5G and H).

After 72 h of siRNA transfection, a significant increase in suspended cells and cell debris could be noticed under the microscope. Consequently, we assessed apoptosis by Annexin V-APC/propidium iodide (PI) staining and flow cytometry showing an enhancement in apoptosis of human SSCs by USP11 siRNA1-2 compared to control siRNA (Fig. 3F and G) and a decrease of their apoptosis by overexpression of USP11 (Fig. S5I and J). Additionally, we examined cell DNA break by USP11 siRNAs using the TUNEL kit. We found that TUNEL-positive cells were significantly increased by USP11 siRNA1-2 compared to control siRNA in human SSCs (Fig. 3H and I). In addition, we examined the influence of USP11 on the cell cycle progression of human SSCs. Western blots indicated that, compared to control siRNA, the levels of cyclin A2, cyclin B1, cyclin H, and CDK2 were diminished by USP11 siRNA1-2 compared to control siRNA in human SSCs (Fig. 3J and K). Taken together, these data implicate that USP11 silencing results in decreases in cell proliferation and DNA synthesis and an increase of apoptosis in human SSCs and influences cell cycle progression.

### HOXC5 is a putative downstream target of USP11 with an interaction in human SSCs

**Fig. 4:**
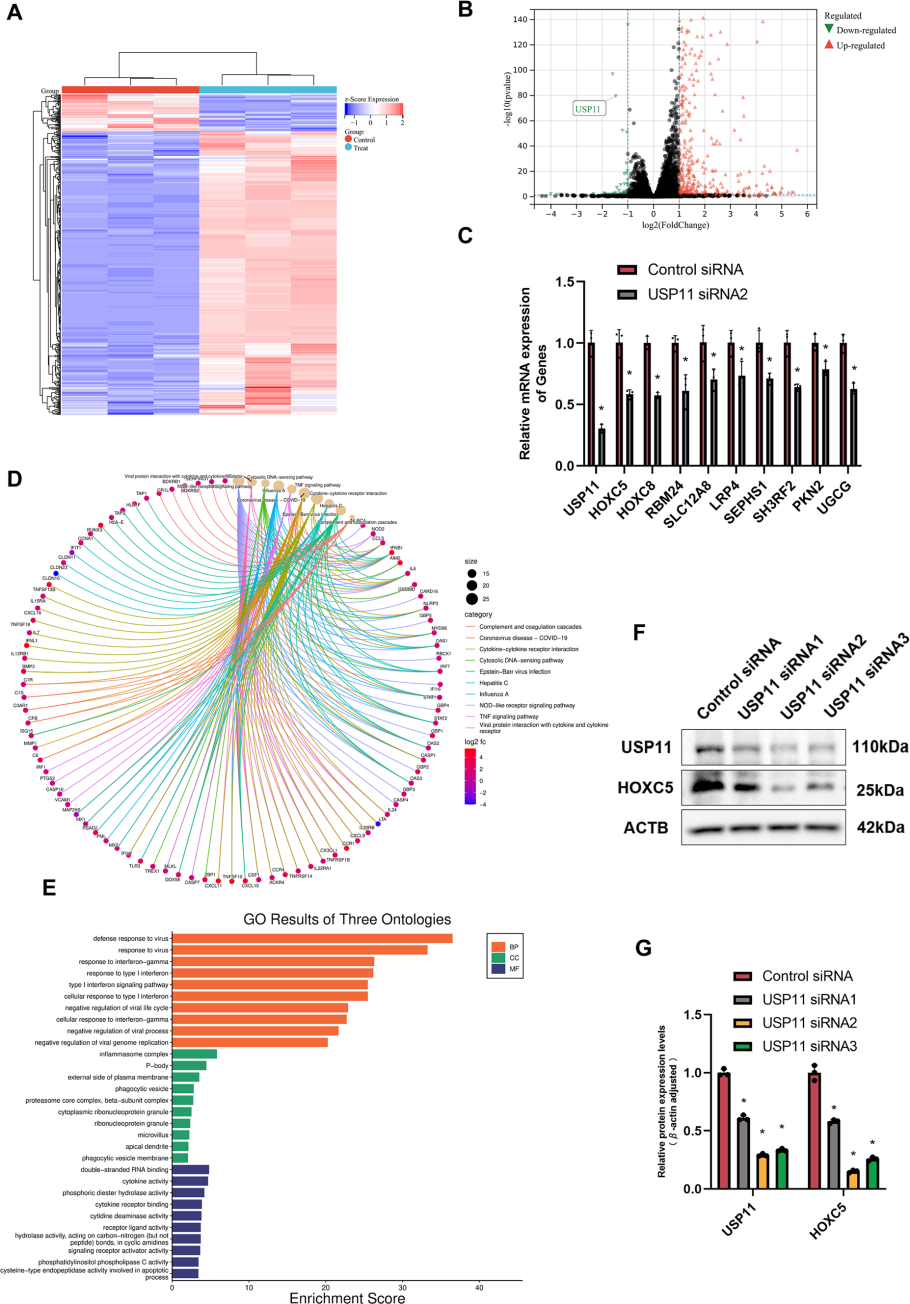
HOXC5 is a downstream target of USP11 in human SSCs. **A**, **B** Heatmap and volcano plot illustrated the differential gene expression profiles in human SSCs between USP11siRNA2 and control siRNA by RNA sequencing. Red denoted the up-regulated DEGs, whereas blue represented the down-regulated DEGs. Control group, control siRNA; Treat group, USP11 siRNA2 treatment. **C** The qPCR was employed to validate the expression profiles of 10 randomly selected DEGs from RNA sequencing data. **D** Gene-Concept Network illustrated the ten key pathways enriched in the KEGG for DEGs. The results of the Gene-Concept Network were visually depicted through nodes and edges, where each node represented a gene or pathway, and the edges between nodes signified their relationships. The size or color of the nodes reflected the magnitude of the fold changes in gene expression. **E** Bar chart illustrated the GO enrichment analysis results of three ontologies for DEGs. **F**, **G** Western blots indicated that USP11siRNA1-3 decreased the protein levels of HOXC5 in human SSCs. “*” denoted a statistically significant difference with a *p*-value < 0.05

To identify the targets of USP11 in regulating the fate determinations of human SSCs, we conducted RNA sequencing using RNA interference (RNAi). After quality control, filtering, and alignment to the reference sequence, a total of 16,629 genes were present in human SSCs without or with USP11 siRNA2 and control siRNA. Based upon selection criteria of |log_2_FC|> 1 and *p* < 0.05, we revealed that there were 479 differentially expressed genes (DEGs) in human SSCs between USP11 siRNA2 and control siRNA. Specifically, 55 genes were significantly down-regulated by USP11 siRNA2, whereas 424 genes were obviously up-regulated by USP11 siRNA2 (Fig. 4A and B). To validate the reliability of RNA sequencing data, we randomly selected 10 DEGs for qPCR analysis. Our qPCR demonstrated that the transcripts of *USP11*, *HOXC5*, *HOXC8, RBM24*, *SLC12A8*,  *LRP4, SEPHS1*, *SH3RF2*, *PKN2,* and *UGCG* were decreased by USP11 siRNA2 in human SSCs (Fig. 4C), which was consistent with RNA sequencing data. Subsequently, we conducted GO and KEGG Pathway analyses on DEGs, which indicated their functions and pathways were primarily involved in biological processes and associated with signaling transduction processes, including protein interaction with cytokine and cytokine receptor, the NOD-like receptor signaling pathway and TNF signaling pathway (Fig. 4D–E). HOXC5 belongs to the homeobox gene family and it serves as a sequence-specific transcription factor with a crucial role in controlling cellular development. Using Western blots, we observed that USP11 silencing resulted in decreases in HOXC5 level in human SSCs (Fig. 4F and G), which implies that HOXC5 may function as a downstream target of USP11 in these cells.

**Fig. 5:**
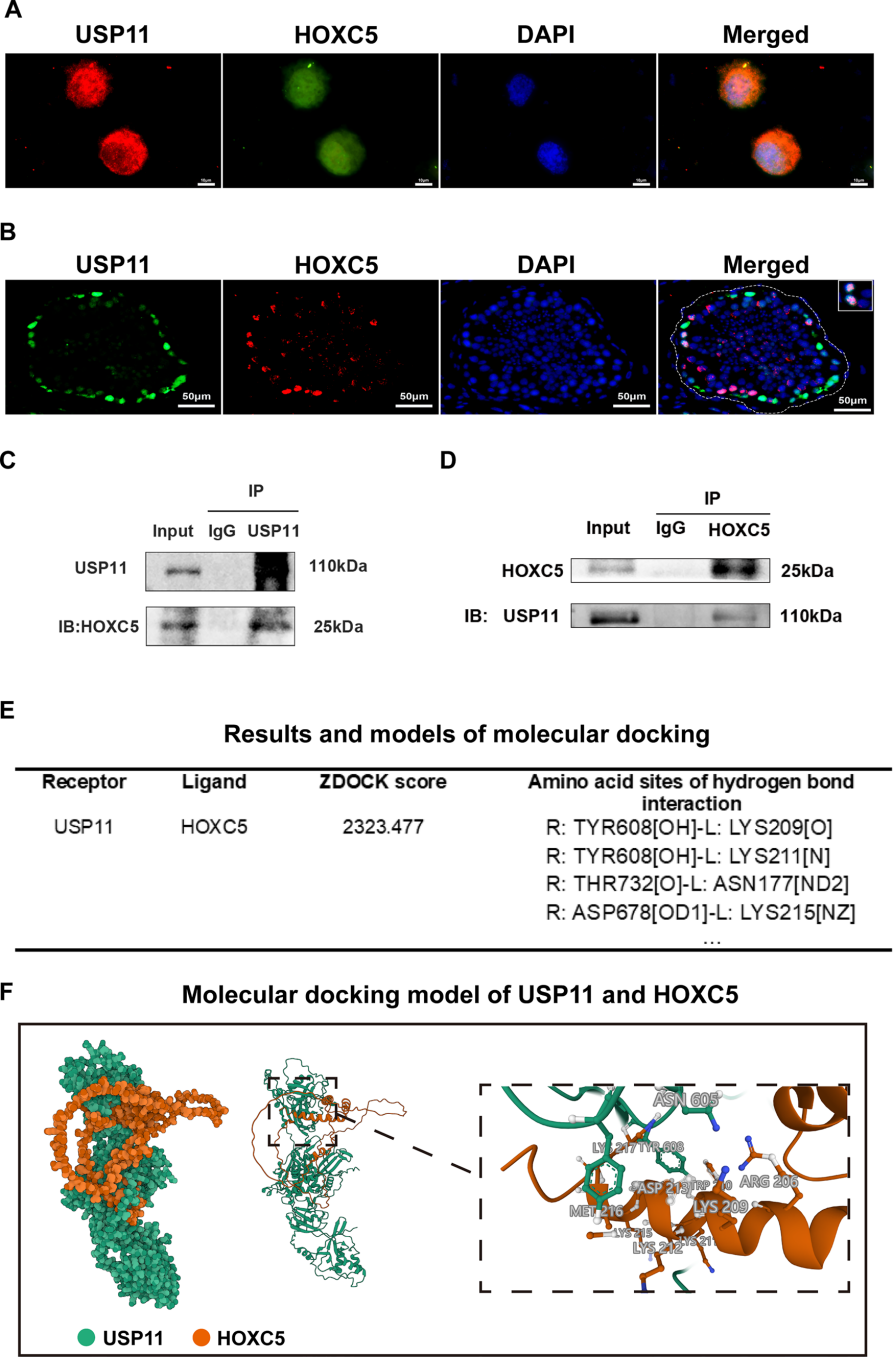
There is an interaction between USP11 and HOXC5 in human SSCs. **A** Dual immunofluorescence illustrated co-localization of USP11 and HOXC5 in human SSCs. Green fluorescence indicated HOXC5-positive cells, while red fluorescence denoted USP11-positive cells. Blue fluorescence represented cell nuclei by DAPI. Scale bars: 10 µm. **B** Double immunofluorescence revealed co-localization of USP11 and HOXC5 in seminiferous tubules of human testes. Green fluorescence indicated USP11-positive cells, while red fluorescence denoted HOXC5-positive cells. Blue fluorescence represented cell nuclei by DAPI. Scale bars: 50 µm. **C**, **D** Co-immunoprecipitation (Co-IP) demonstrated the interaction between USP11 and HOXC5 in human SSCs. **E** Molecular docking between USP11 and HOXC5 in human SSCs. TYR608 represented tyrosine at the 608th position in the peptide chain. Notes: *L* ligand, *R* receptor, *TYR* tyrosine, *LYS* lysine, *ASN* asparagine, *THR* threonine, *ASP* aspartic acid. **F** Molecular docking model of USP11 and HOXC5. Rigid protein–protein docking (ZDOCK) was performed between USP11 and HOXC5 to examine their relationships. The crystal structures of USP11 and HOXC5 were derived from the AlphaFold Protein Structure Database. The model with the highest Z-score was selected from the prediction results for presentation. Specific amino acid residues were involved in protein–protein interaction using a ball-and-stick model. USP11 was shown in green, while HOXC5 was indicated in orange. The dashed box indicated an enlarged display of the specific region where USP11 and HOXC5 were interacted

In order to clarify the reciprocal relationship between USP11 and HOXC5, we performed double immunofluorescence, Co-IP, and molecular docking assays. Double immunofluorescence illustrated the co-localization of USP11 and HOXC5 in the cell nuclei and cytoplasm of human SSCs (Fig. 5A) and in normal human testicular tissues (Fig. 5B). Our Co-IP assay demonstrated that USP11 successfully pulled down HOXC5 in human SSCs (Fig. 5C), and reciprocally, HOXC5 effectively precipitated USP11 in these cells (Fig. 5D), which indicates mutual interaction between these two proteins. Furthermore, at the molecular level, molecular docking elucidated an interaction between USP11 and HOXC5. As illustrated in Fig. 5E, the optimal model obtained by ZDOCK had a score of 2323.477, while visual analysis of molecular docking revealed that USP11 could establish hydrogen binding interactions with amino acid sites, e.g., TYR608-LYS209 and TYR608-LYS211 (Fig. 5F), which facilitates their stable binding. Considered together, these results indicate a significant interaction between USP11 and HOXC5 in human SSCs.

### HOXC5 knockdown suppresses proliferation and DNA synthesis and elevates apoptosis of human SSCs

**Fig. 6:**
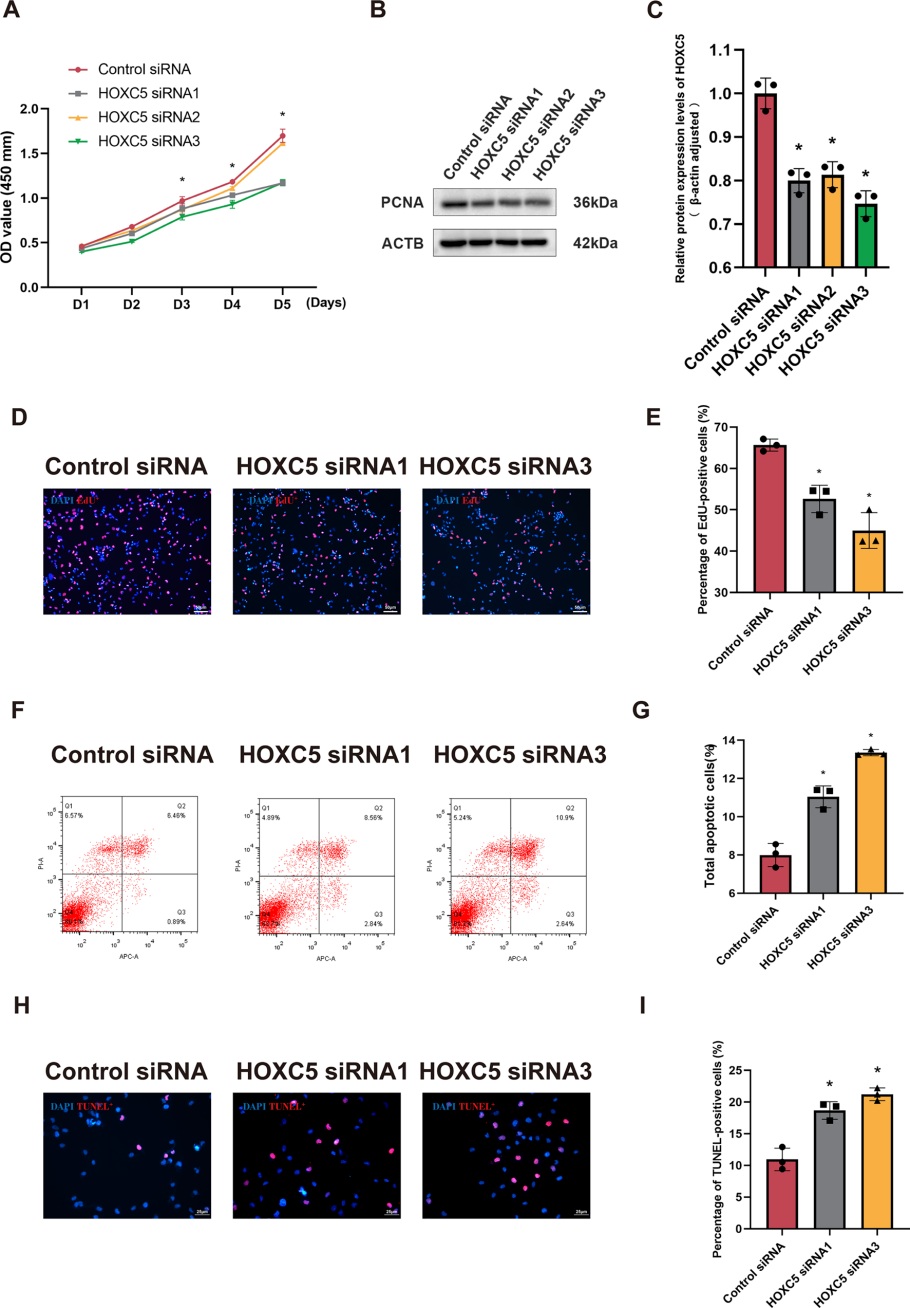
Influence of HOXC5 silencing on proliferation and apoptosis of human SSCs. **A** CCK-8 assay showed proliferation of human SSCs by HOXC5 silencing. **B**, **C** PCNA expression levels were measured by Western blots in human SSCs after HOXC5 silencing. **D**, **E** EdU assay indicated DNA synthesis in human SSCs after HOXC5 silencing. EdU-positive cells were  visualized by red fluorescence. Scale bars: 50 µm. **F**, **G** Cellular apoptosis of human SSCs after HOXC5 silencing was assessed by flow cytometry. **H**, **I** TUNEL assay was employed to determine apoptosis of human SSCs by HOXC5 silencing. TUNEL-positive cells were stained in red fluorescence. Scale bars: 25 µm. “*” denoted a statistically significant difference with a* p*-value < 0.05

To further explore the function of HOXC5 in mediating human SSC determinations, we designed three pairs of siRNAs targeting *HOXC5*. HOXC5 siRNA1-3 decreased transcript and protein of HOXC5 in human SSCs (Fig. S6). Compared to control siRNA, CCK-8 assay showed an obvious decrease in the number of human SSCs at day 3 after HOXC5 siRNA1-3 treatment (Fig. 6A). Western blots demonstrated a significant reduction in the expression level of PCNA in human SSCs following HOXC5 siRNA1-3 treatment (Fig. 6B and C). Concurrently, EdU assay revealed obvious inhibition in the proportion of EdU-positive cells in human SSCs with HOXC5 siRNA1 and 3 (Fig. 6D and E). Subsequently, we explored the impact of interfering with the expression of HOXC5 on apoptosis in human SSCs through flow cytometry and TUNEL assay. Flow cytometry showed an obvious increase in apoptotic cells in human SSCs with HOXC5 siRNA1 and HOXC5 siRNA3 (Fig. 6F and G). TUNEL assay revealed a marked elevation in the number of TUNEL-positive cells in human SSCs by HOXC5 siRNA1,3 compared to control siRNA (Fig. 6H and I). Based on the aforementioned findings, we conclude that HOXC5 silencing inhibits proliferation and DNA synthesis of human SSCs and enhances apoptosis of these cells.

### The biological effect of USP11 downregulation on human SSCs can be reversed by the overexpression of HOXC5

**Fig. 7:**
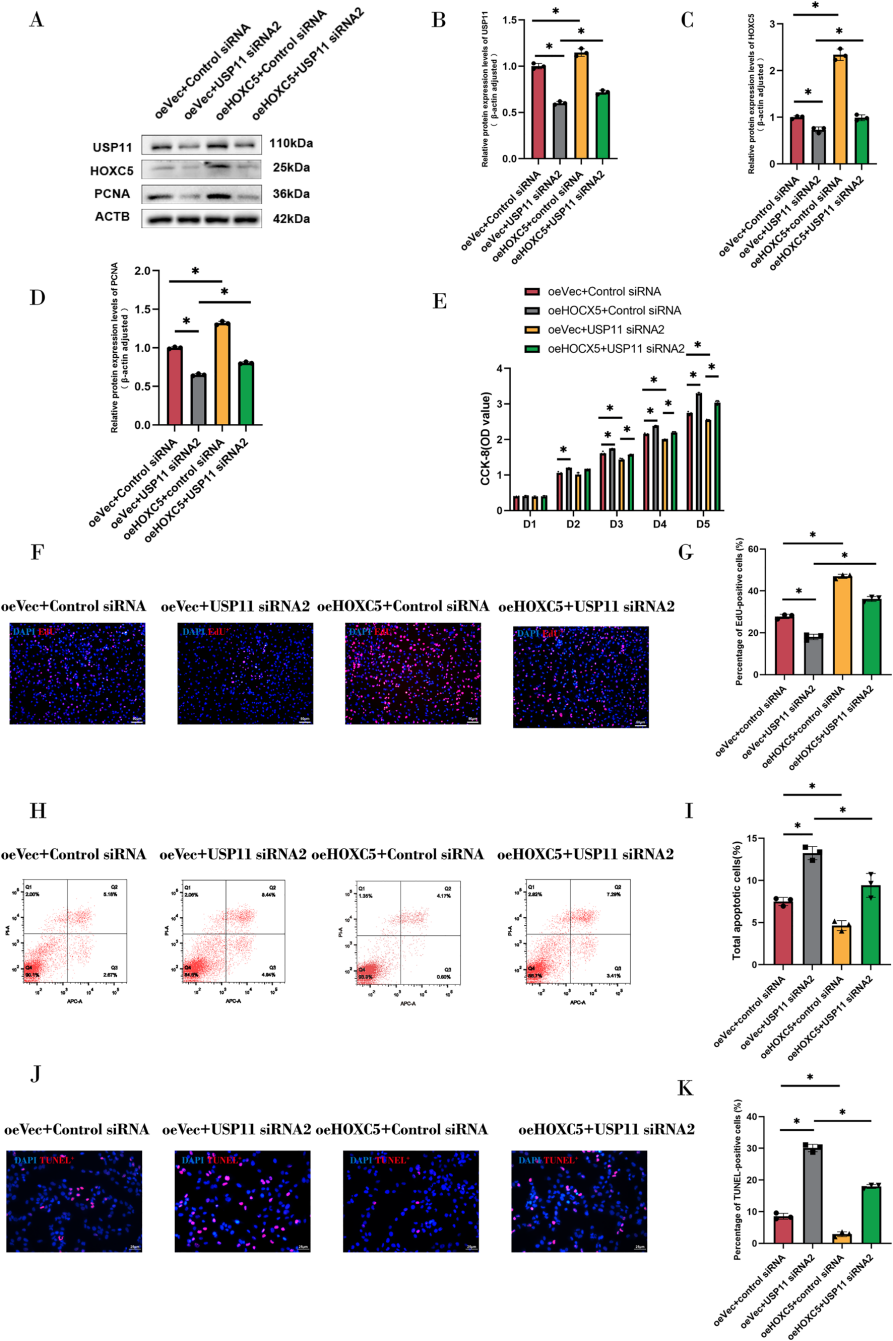
Functional rescue assay by USP11 silencing in human SSCs overexpressing HOXC5. **A**–**D** Expression levels of USP11, HOXC5, and PCNA were detected in human SSCs with USP11 siRNA2 and HOXC5 overexpressing. **E** CCK-8 assay assessed proliferation in HOXC5 overexpressing SSCs after USP11 siRNA2 treatment. **F**, **G**. EdU assay examined DNA synthesis in HOXC5 overexpressing SSCs after USP11 siRNA2 treatment. Red fluorescence indicated EDU-positive cells. Scale bars: 50 µm. **H**, **I** Cellular apoptosis in HOXC5 overexpressing SSCs after USP11 siRNA2 treatment  as detected by flow cytometry. **J**, **K** TUNEL assay detected cellular apoptosis in HOXC5 overexpressing SSCs after USP11 siRNA2 treatment. Red fluorescence represented TUNEL-positive cells. Scale bars: 25 µm. “*” denoted a statistically significant difference with a *p*-value < 0.05

We asked whether the biological functions of USP11 in human SSCs were associated with HOXC5, and we conducted functional rescue experiments by knocking down USP11 in human SSCs overexpressing HOXC5. Initially, we successfully established a stable human SSC line overexpressing HOXC5, as shown by qPCR and Western blots (Fig. S7A–D). Western blots demonstrated that overexpression of HOXC5 reversed the downregulation of PCNA in human SSCs caused by USP11 silencing (Fig. 7A–D). CCK-8 assay indicated that overexpression of HOXC5 attenuated the inhibitory effect of USP11 knockdown on the proliferation of human SSCs (Fig. 7E). Similarly, EdU incorporation assay demonstrated that overexpression of HOXC5 significantly alleviated inhibitory influence of USP11 siRNA2 on DNA synthesis (Fig. 7F and G). Flow cytometry and TUNEL assay revealed that the overexpression of HOXC5 rescued USP11 knockdown-induced apoptosis in human SSCs (Fig. 7H–K). Collectively, these data imply that overexpression of HOXC5 can rescue proliferation, DNA synthesis, and apoptosis caused by USP11 knockdown in human SSCs.

### HOXC5 mediates fate decisions of human SSCs via the canonical WNT/β-catenin signaling pathway

**Fig. 8:**
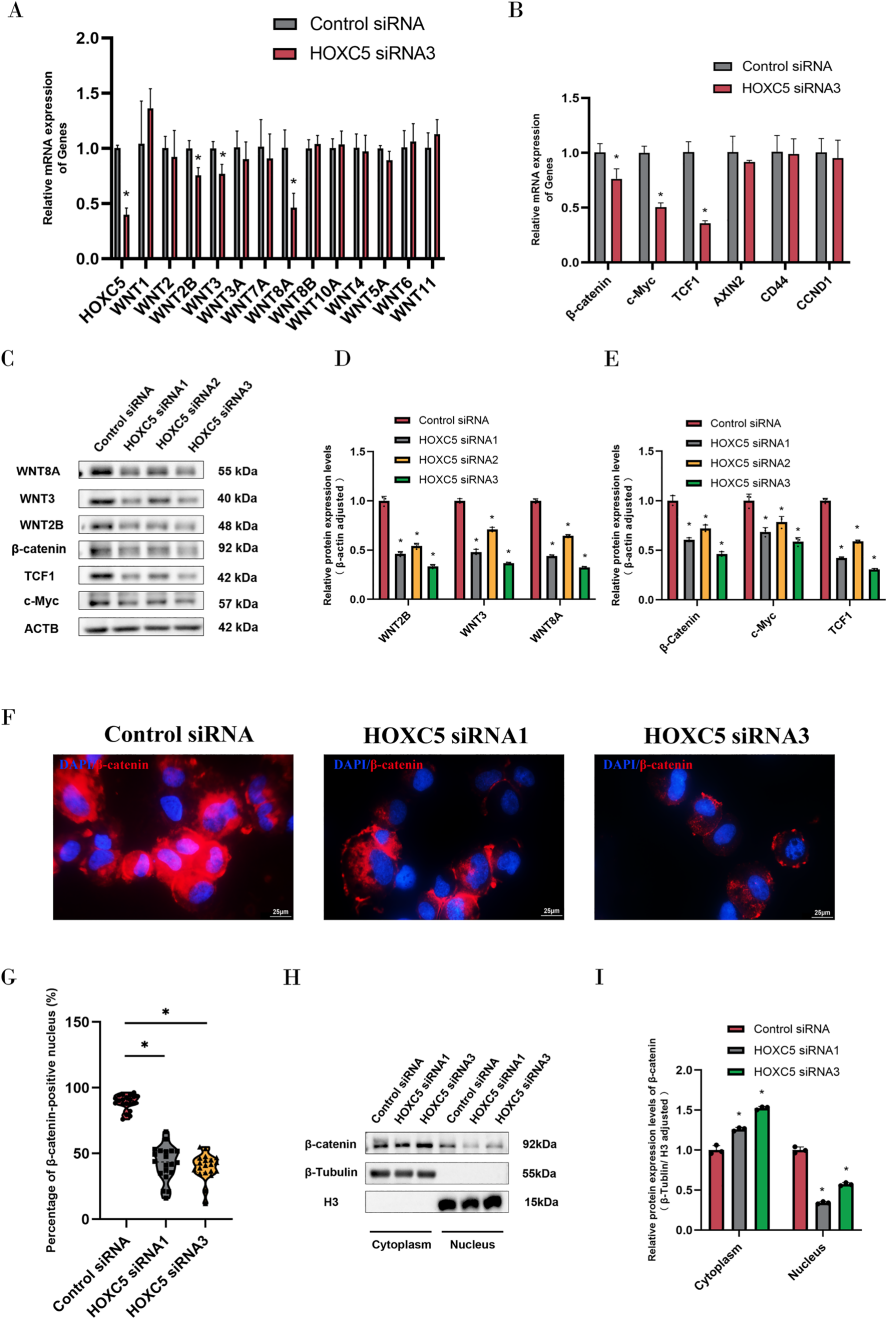
HOXC5 regulates human SSC fate decisions through the canonical WNT signaling pathway. **A** Transcription levels of classical *WNT* family genes were detected by qPCR in human SSCs after HOXC5 silencing. **B** The mRNA expression of β-catenin-related target genes in human SSCs after HOXC5 silencing was assayed by qPCR. **C**–**E** Protein expression levels of WNT2B, WNT3, WNT8A, and β-catenin-related target proteins were detected by Western Blotting after silencing HOXC5. **F**, **G** Nuclear translocation of β-catenin was detected by immunocytochemistry in human SSCs after HOXC5 knockdown. Red fluorescence represented β-catenin-positive cells. Scale bars: 25 µm. **H**, **I** Protein expression levels of β-catenin in the cytoplasm and nuclei of human SSCs after HOXC5 knockdown were measured by nuclear-plasmic separation assay. “*” denoted a statistically significant difference with a *p*-value < 0.05

WNT signaling pathway is an intricate pathway that is essential for different biological processes, including tissue homeostasis, cell fate determinations, and embryonic development [30]. It has been shown that HOXC5 plays a role in lung development by modulating the WNT signaling pathway [31]. Therefore, we hypothesized that HOXC5 regulates the fate decisions of human SSCs through the WNT signaling pathway. To test this hypothesis, we utilized qPCR to examine the transcription levels of common WNT family genes in human SSCs after silencing HOXC5. We observed a significant downregulation in mRNA expression levels of some genes associated with the classical WNT signaling pathway, e.g., *WNT2B, WNT3,* and *WNT8A*, in human SSCs by silencing HOXC5 (Fig. 8A). Subsequently, we assessed mRNA expression of β-catenin and its target genes, and we revealed that HOXC5 knockdown suppressed the transcription of *β-catenin*, *TCF1*, and *c-Myc* (Fig. 8B). Simultaneously, Western blots demonstrated that the levels of WNT2B, WNT3, WNT8A, β-catenin, TCF1, and c-Myc were decreased by HOXC5 siRNA1-3 (Fig. 8C–E). Immunofluorescence illustrated a reduction in the nuclear translocation of β-catenin in human SSCs by HOXC5 knockdown (Fig. 8F and G). Our nuclear-cytoplasmic fractionation experiments further showed a significant decrease in the expression levels of β-catenin in the cell nuclei of human SSCs due to the downregulation of HOXC5 (Fig. 8H and I). Taken together, these findings suggest that HOXC5 modulates the fate determinations of human SSCs through the classical WNT/β-catenin signaling pathway.

### Low expression levels of USP11 are relevant to spermatogenesis defects

**Fig. 9:**
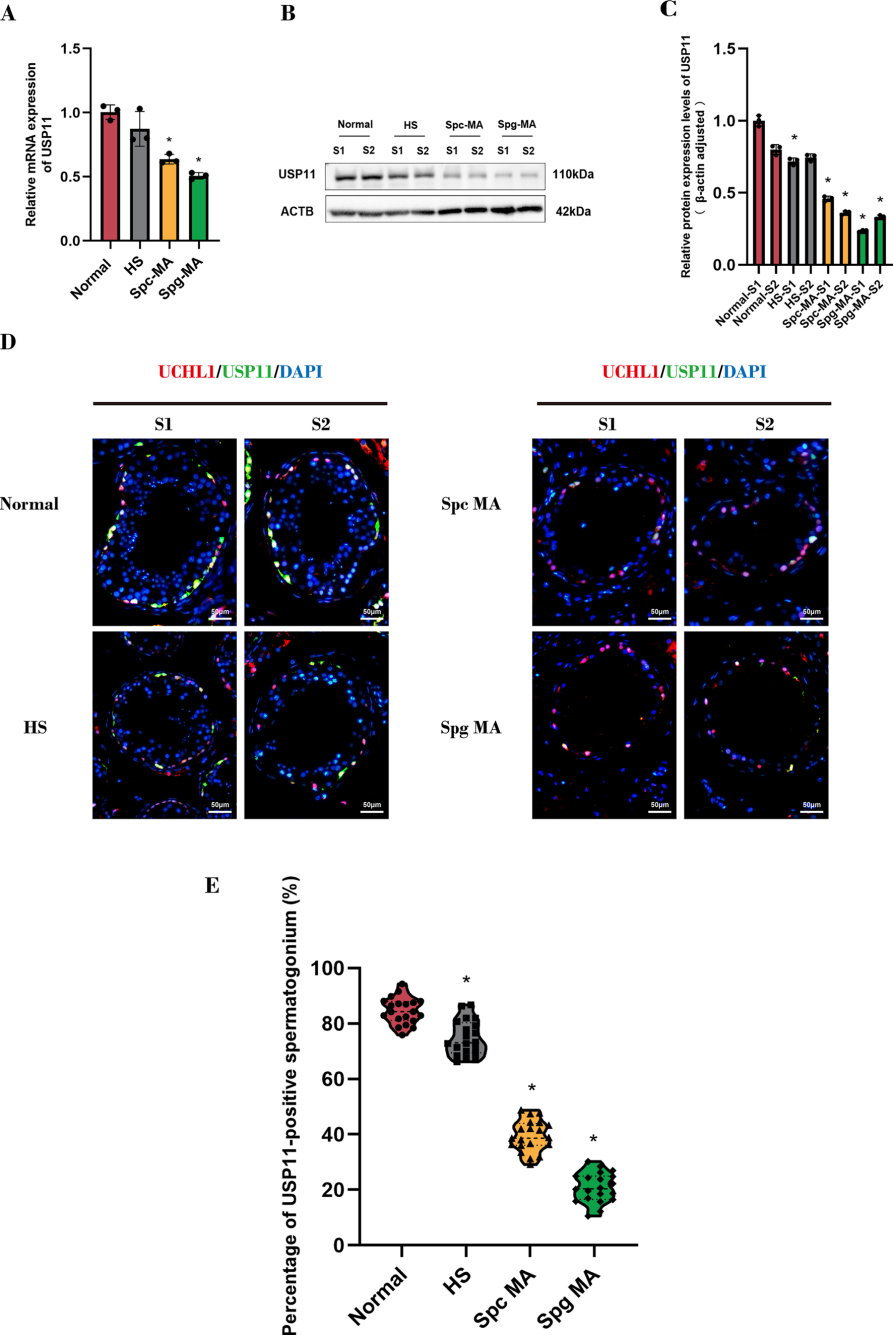
The expression profiles of USP11 in human testicular tissues with spermatogenic failure. **A** The mRNA levels of *USP11* in human testicular tissues of various spermatogenic disorders were detected by qPCR. **B**, **C** Western blots demonstrated protein expression levels of USP11 in human testicular tissues with spermatogenic failure. **D** The cellular localization of USP11 in human testicular tissues with spermatogenesis disorders was assessed by double immunofluorescence. Green fluorescence indicated USP11 signals, and red fluorescence represented UCHL1 signals. DAPI was used to label cell nuclei. Scale bars: 50 µm. **E** The percentages of USP11-positive spermatogonia in human testicular tissues with spermatogenesis disorders were displayed by violin plot. “*” denoted a statistically significant difference with a *p*-value < 0.05

To explore whether abnormal expression of USP11 is associated with spermatogenesis disorder, testicular tissues were obtained from NOA patients undergoing testicular biopsy. Based upon histopathological examination and Johnsen scoring, these tissues were classified into four categories, including normal spermatogenesis (Normal), hypospermatogenesis (HS), spermatogenic arrest at the spermatocyte maturation stage (Spc MA), and spermatogenic arrest at the spermatogonial maturation stage (Spg MA) (Fig. S8). Subsequently, total RNA and protein were extracted from testicular tissues for further analysis. Our qPCR indicated a lower level of* USP11* transcription in both Spc MA and Spg MA (Fig. 9A). Simultaneously, Western blots revealed a significant decrease in USP11 protein levels in testis tissues of HS, Spc MA, and Spg MA (Fig. 9B and C). Double immunofluorescence illustrated that there was a notable decrease in the percentages of USP11-positive cells within seminiferous tubules of Spc MA and Spg MA (Fig. 9D and E). Together, these data reflect that a lower level of USP11 is associated with spermatogenesis failure.

## Discussion

SSCs, as the initiating cells of spermatogenesis, are essential for maintaining sperm production throughout the entire lifespan [9, 10], and they can be used to restore male fertility for patients with cancer [21, 22]. Therefore, it is of unusual significance to explore molecular mechanisms regulating the fate decisions of human SSCs. In this study, we have uncovered for the first time a novel mechanism by which USP11 regulates the fate determinations of human SSCs. We have demonstrated that USP11 silencing suppressed the proliferation and DNA synthesis of human SSCs and enhanced their apoptosis, and we identified HOXC5 as a downstream target of human SSCs. Furthermore, we have revealed that HOXC5 modulated the fate decisions of human SSCs through mediating the canonical WNT/β-catenin signaling pathway. Importantly, our findings implicate a potential association between aberrant expression levels of USP11 and spermatogenesis disorders.

USP11 belongs to the family of ubiquitin-specific proteases (USP) deubiquitinase, and it has been found to exhibit abnormal expression in various human malignancies [32]. USP11 functions as a tumor suppressor by stabilizing VGLL4 in a YAP-dependent manner, which inhibits cancer cell growth, migration, and invasion [33]. On the other hand, USP11 significantly stimulates proliferation and metastasis of hepatocellular carcinoma cells both in vitro and in vivo [34]. Additionally, USP11 interacts with XIAP to suppress apoptosis in breast cancer cells [35]. USP11 regulates cell cycle progression and DNA damage response in lung cancer cells in a p21-dependent manner [36]. These studies implicate that USP11 plays an important role in controlling cell fate determinations. In this study, we demonstrated that USP11 silencing led to decreases in proliferation and DNA synthesis and an enhancement in apoptosis of human SSCs. We also found that USP11 knockdown resulted in a reduction in levels of cell cycle-related proteins, including cyclin A2, cyclin B1, cyclin H, and CDK2 in these cells.

To further investigate the specific mechanisms by which USP11 regulates the fate decisions of human SSCs, we conducted RNA sequencing on SSCs treated with USP11 siRNAs. Interestingly, our RNA sequencing revealed that transcripts of *HOXC* gene cluster, including *HOXC4*, *HOXC5*, *HOXC8*, and *HOXC9* (Supplementary Table 5), were decreased by USP11 siRNAs. This suggests a potentially significant role for the HOXC family in regulating fate determinations of human SSCs. Due to the most pronounced down-regulation of HOXC5 by USP11, we focused on exploring the function and signaling of HOXC5 in human SSCs.

HOX genes belong to the homeobox transcription factor family, and human HOX genes are classified into four clusters (HOXA, HOXB, HOXC, and HOXD), with HOXC4, HOXC5, and HOXC6 being members of HOXC gene cluster [37, 38]. HOXC4 functions as a GDNF-regulated gene and it modulates the stem cell activity of rat SSCs [39]. Additionally, HOXC homeobox gene cluster (HOXC4, HOXC6, HOXC8, and HOXC9) can be activated by DOT1L (H3K79 methyltransferase), which maintains self-renewal of mouse SSCs [40]. In this study, we observed that the knockdown of HOXC5 gene resulted in a deceleration of proliferation, reduced DNA synthesis, and an increased number of apoptotic cells in human SSCs. Subsequently, we found that the protein levels of HOXC5 were decreased concomitantly by USP11 silencing. Significantly, we found that USP11 could interact with HOXC5 in human SSCs. Dual immunofluorescence of human testicular tissues and human SSCs revealed co-localization of HOXC5 and USP11, implying potential interactions between these two proteins. Our Co-IP and molecular docking experiments further demonstrated the interaction between HOXC5 and USP11 in human SSCs.

WNT signaling pathway constitutes a highly complex biological regulatory network, including WNT/β-catenin pathway, WNT-PCP pathway, and WNT-Ca^2^^+^ pathway [30]. As a traditional WNT pathway, WNT/β-catenin pathway is dependent on β-catenin as its primary functional effector, and it is essential for controlling cell division [30, 41]. Ascorbic acid modulates endogenous ROS levels through the WNT/β-catenin signaling pathway in a dose-dependent manner, thereby affecting the proliferation of SSCs [42]. We found that HOXC5 silencing results in decreases in the transcription of common *WNT* family genes in classical pathways, e.g., *WNT2B, WNT3*, and *WNT8A*. Simultaneously, transcripts of *β-catenin* and its target genes (*TCF1, c-Myc*) were reduced by HOXC5 silencing. Therefore, we speculated that the classical WNT/β-catenin pathway is involved in the regulation of HOXC5 in proliferation and apoptosis of human SSCs. Immunofluorescence and nuclear-cytoplasmic fractionation experiments demonstrated that HOXC5 influences the nuclear translocation of β-catenin. Taken together, our results indicate that HOXC5 participates in regulating the fate decisions of human SSCs by mediating the classical WNT/β-catenin signaling pathway. Moreover, we observed a correlation between lower levels of USP11 expression and NOA patients with spermatogenesis disorders.

**Fig. 10:**
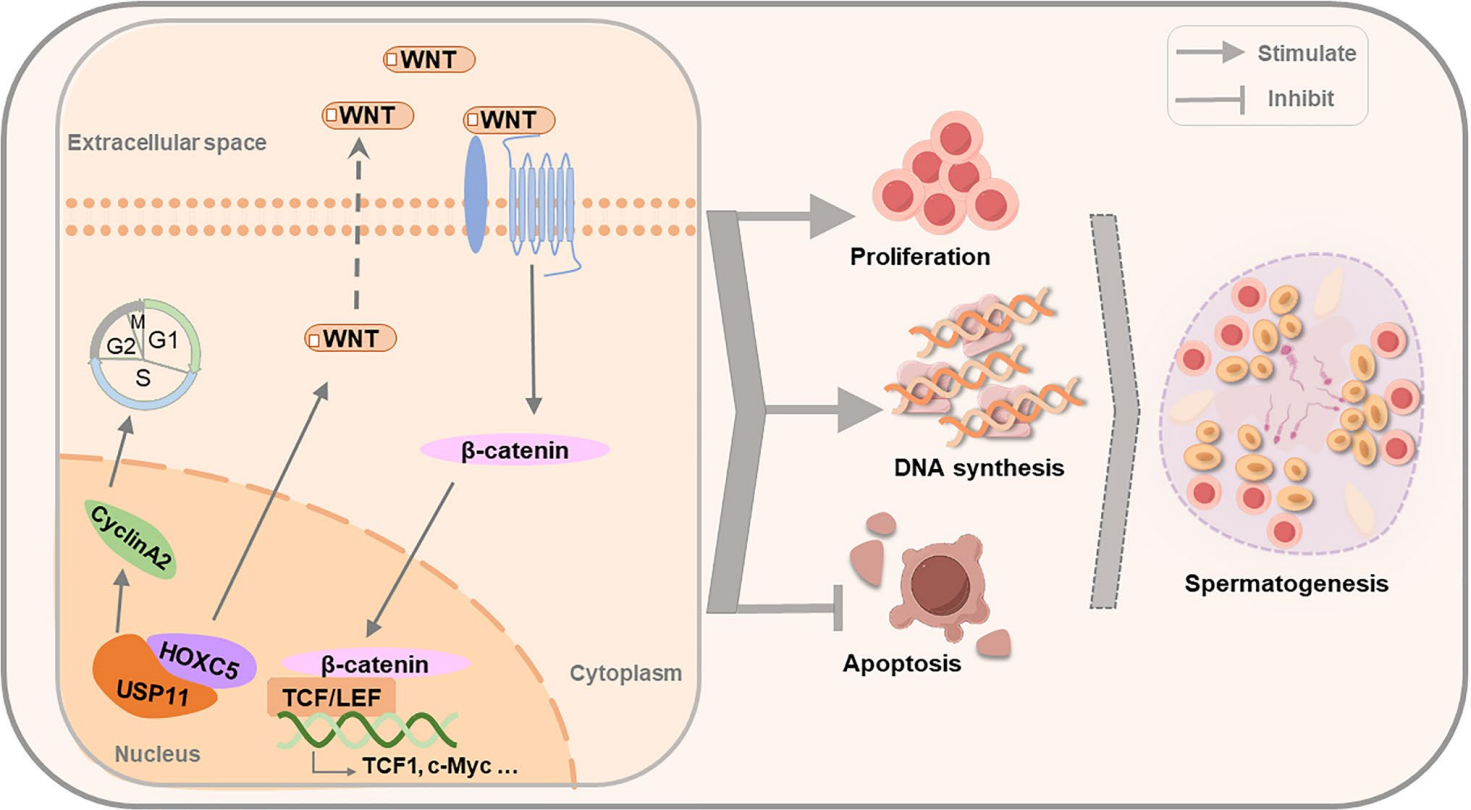
The function and mechanism of USP11 in controlling the proliferation, DNA synthesis, and apoptosis of human spermatogonial stem cells. USP11 interacts with HOXC5 to regulate the fate decisions of human SSCs via the classical WNT/β-catenin signaling pathway

In summary, we have unveiled the predominant expression of USP11 in human SSCs. We are the first to report that USP11 controls the proliferation and apoptosis of human SSCs. We have identified HOXC5 as a target of USP11, and we revealed a mutual interaction between USP11 and HOXC5 in human SSCs (Fig. 10). Furthermore, we demonstrated that USP11/HOXC5 regulates the fate decisions of human SSCs by mediating the classical WNT/β-catenin signaling pathway (Fig. 10). This study thus provides a novel molecular mechanism governing the fate of human SSCs and provides new targets for gene therapy of male infertility.

## Materials and methods

### Analysis of single-cell sequencing datasets from GSE109037, GSE112013 and GSE134144 datasets

GSE112013, GSE149512, and GSE231 datasets were obtained and downloaded from the GEO website (https://www.ncbi.nlm.nih.gov/gds), and data processing was performed using Seurat (version 4.3.0). Initially, expression matrix data was imported into R using the Read10X, resulting in the creation of three Seurat objects. Data filtering was conducted based on the criteria of cell gene expression levels falling between 500 and 10,000, and mitochondrial gene proportions were less than 20%. The NormalizeData and FindVariableFeatures functions were applied to each Seurat object using default settings. A selection of the top 2,000 highly variable genes and the first 20 principal components (PC) was completed. Subsequently, dimensionality reduction and clustering analysis were conducted on the processed Seurat objects using uniform manifold approximation and projection (UMAP), and clusters were annotated using marker genes. FindMarkers command was employed to identify differentially expressed genes (DEGs) between SSCs and other male germ cells. Using the ggvenn package (version 0.1.10), we obtained the intersection genes from these three datasets of DEGs. Bubble plots and violin plots were created and modified using ggplot2 (version 3.44).

### Acquisition of human testicular tissues

Human testicular tissues used in this study were obtained from patients undergoing prostate cancer surgery with castration therapy at the Xiangya Third Hospital of Central South University or microdissection testicular sperm extraction (m-TESE) at the Reproductive & Genetic Hospital of CITIC-Xiangya. The tissues were cleaned with PBS containing antibiotics, and they were either fixed in Bouin's fixative or stored in liquid nitrogen for future studies. All specimens were collected post-clinical treatment, and all participating patients were fully informed about the study and provided signed informed consent. This study was approved by IRB of the Third Xiangya Hospital of Central South University (Approval No: 22131).

### Culture of human SSC line

The human SSC line used in this study was established by overexpressing SV40 large T antigen in human primary GPR125-positive undifferentiated spermatogonia (SSCs). This cell line expressed numerous SSC markers, including GPR125, GFRA1, PLZF, UCHL1, and THY1 (Fig. S1), and it exhibited phenotypic characteristics similar to human primary SSCs. These cells were cultured under the conditions of 34 °C and 5% CO_2_ incubator with DMEM/F12 medium (Gibco, USA) containing 1% penicillin–streptomycin (Gibco, USA) and 10% fetal bovine serum (Gibco, USA).

### Construction of overexpressing stable cell lines and gene silencing

All of the overexpression plasmids of genes were provided by MiaoLingBio (Wuhan, China). Following plasmid transformation and amplification, the target plasmid and the second-generation lentiviral packaging plasmids (Target plasmid: PsPAX2: PMD2.G = 3:2:1) were co-transfected into 293T cells using Lipofectamine 3000 transfection reagent (Invitrogen, CA, USA) in terms of manufacturer’s instructions. Subsequently, a cell culture supernatant containing virus particles was collected and concentrated, and it was used to infect human SSCs. Stably overexpressing cell lines of genes were obtained after selection with puromycin (1 μg/ml). The overexpression effect was confirmed through qPCR and Western blots.

The small interfering RNAs (siRNAs) utilized in this study were obtained from Tsingke Biotech (Beijing, China), and siRNAs were transfected into human SSCs using Lipofectamine 3000 (Invitrogen, CA, USA). RNA was isolated within 48 h post-transfection, while protein extraction was conducted at 72 h post-transfection. All siRNA sequences were detailed in Supplementary Table 1.

### Reverse transcription (RT) PCR and real-time quantitative PCR

Total RNA was extracted from testicular tissues or cells using RNA isolater Total RNA Extraction Reagent (R401-01, Vazyme Biotech, Jiangsu, China) in terms of manufacturer's instructions, and the quality and concentration of the extracted RNA were assessed using Nanodrop (Thermo Fisher Scientific, USA). The extracted RNA displayed OD_260_/OD_280_ ratios ranging from 1.8 to 2.1. Subsequently, reverse transcription (RT) was performed using Evo M-MLV RT Premix reagent (AG11706, Accurate Biotechnology, Hunan, China) to obtain cDNA. PCR reactions were performed according to the instructions of Accurate Biotechnology (Hunan, China) for 2X Accurate Taq Master Mix (dye plus) II (AG11022) with the Applied Biosystems MiniAmp thermal cycler (Thermo Fisher Scientific, USA). Subsequently, electrophoresis of the PCR products was performed on 2% agarose gels, and images were acquired using the ChampGel™ 5000 gel imaging system (Beijing, China).

For real-time PCR, a 20 μl reaction system was prepared using the qPCR Kit (AG11701, Accurate Biotechnology, Hunan, China). Real-time qPCR was performed on the CFX Connect™ Fluorescent Quantitative PCR Detection System (Bio-Rad, USA) using a two-step PCR amplification method. The relative expression levels of the target genes in the treatment and control groups were calculated using the formula 2^−ΔΔCt^ [ΔΔCt = ΔCt (treatment group)−ΔCt (control group)]. The primers used were supplied by Sangon Biotech (Shanghai, China), and the detailed information was showed in Supplementary Table 2.

### Western blots, immunoprecipitation (IP) and Co-IP

Cells or testicular tissues were lysed using RIPA lysis buffer (P0013B, Beyotime, Shanghai, China). The lysates were centrifuged at 12,000 rpm for 20 min at 4 °C, and the supernatant was collected for subsequent experiments. SDS-PAGE gels with appropriate concentrations of proteins were prepared based on the molecular weight of the proteins, and each well was loaded with 25 μg of total proteins. Proteins were transferred onto PVDF membranes (IPVH00010, Millipore, USA), and membranes were incubated with primary antibodies overnight at 4 °C and followed by 1 h incubation with the secondary antibodies at room temperature. Detection of proteins was carried out using a chemiluminescence imaging system (MiniChemi 610, SINSAGE, Beijing, China). The primary and secondary antibodies used in the study were listed in Supplementary Table 3.

Immunoprecipitation (IP) was conducted pursuant to the manual of manufacture. Fresh protein lysate supernatant was obtained, and the appropriate amount of antibody was incubated with the supernatant overnight at 4 °C. Subsequently, BeyoMag™ Protein A + G Magnetic Beads (P2108, Beyotime, Shanghai, China) were added and incubated at room temperature for 1 h to capture the formed complexes. After elution and magnetic separation, the supernatant was collected for Western blots.

### Immunohistochemistry and immunocytochemistry

The paraffin sections of human testis tissues were initially pretreated at 65 °C for 30 min, followed by deparaffinization with xylene and hydration with a graded series of ethanol. Antigen restoration was performed by high-temperature boiling in citrate antigen retrieval solution (BL619A, biosharp, Anhui, China). The testis sections were treated with Endogenous Peroxidase Blocking Buffer (P0100A, Beyotime, Shanghai, China) for 15 min, blocked with 5% BSA at room temperature for 1 h, and then incubated with the primary antibodies overnight at 4 °C. After washing three times with PBS, the sections were incubated with the corresponding secondary antibodies at room temperature for 1 h and followed by DAB staining. For tissue immunofluorescence, the sections were incubated with the appropriate Alexa Fluor-conjugated secondary antibodies at room temperature for 1 h after washing with PBS. After nuclear staining with DAPI for 8 min, immunofluorescence was observed using a Leica fluorescence microscope (DM3000, Germany).

For immunocytochemistry, a similar procedure to immunohistochemistry was employed. For proteins present intracellularly, 0.25% Triton X-100 treatment was applied for 15 min before antigen blocking to permeabilize cells. All antibodies used in this study could be found in Supplementary Table 4.

### CCK-8 and EdU incorporation assays

Cells were plated at a density of 20,000 cells per well in a 96-well cell culture plate. A mixture of Cell Counting Kit-8 reagent (CCK-8) (K1018, APExBIO, USA) and DMEM/F12 medium in a 1:9 ratio was added to each well through a medium exchange, with a volume of 100 μl per well. After incubation for 3.5 h in a cell culture incubator, the absorbance at 450 nm was measured using the Synergy 2 Multimode Microplate Reader (Biotek, USA). Continuous monitoring of cell growth was conducted for 5 days.

For the 5-Ethynyl-2′-deoxyuridine (EdU) incorporation assay, the culture medium in a 96-well cell culture plate was replaced the day before detection. Cells were then incubated in DMEM/F12 medium containing 0.05% EdU reagent A (C10310-1, RiboBio, Guangzhou, China) for 12 h. Subsequently, cells were fixed with PBS containing 4% paraformaldehyde (PFA) at room temperature for 0.5 h. After multiple washes with PBS containing 0.5% Triton X-100, cells were incubated with 1X Apollo staining reaction mixture in the dark at room temperature for 0.5 h. Following DAPI staining of cell nuclei, EdU-positive cells were observed under a Leica fluorescence microscope (DMi8, Germany).

### Annexin V-APC/PI staining and flow cytometry and TUNEL assay

Annexin V-APC/PI staining was performed according to the instructions of BioLegend (640932, USA). Cells were washed with PBS and resuspended in 100 μl of Annexin V Binding Buffer. Subsequently, each sample was treated with 5 μl APC Annexin V and 10 μl Propidium Iodide (PI) Solution and followed by incubation at room temperature in the dark for 15 min. The reaction was terminated by adding 400 μl Annexin V Binding Buffer. Finally, flow cytometry (FACSCanto II 488N, BD Bioscience, USA) was employed for the detection of apoptosis of cells.

TUNEL assay was conducted using the One-step TUNEL Cy3 Apoptosis Detection Kit (K1134, APExBIO, USA). In brief, cells in a 96-well plate were fixed with 4% PFA  for 25 min, and permeabilized using 0.5% Triton X-100 for 20 min or 20 μg/ml proteinase K for 4–6 min. Subsequently, cells were incubated with the labeling reaction mixture containing Cy3-dUTP at room temperature in the dark for 1 h. After DAPI nuclear counterstaining for 8 min, images were captured by a fluorescence microscope (DMi8, Germany).

### Cytoplasmic and nuclear protein isolation assay

The Specialized Protein Extraction Kit (P0027, Beyotime, Shanghai, China) was utilized for extracting nuclear and cytoplasmic proteins. Initially, we collected precipitates of human SSCs and sequentially added the appropriate amount of Cell Lysis Buffer A and Cell Lysis Buffer B in terms of the kit instructions. After centrifugation at 16,000 g for 5 min at 4 °C, the supernatant was obtained as cytoplasmic protein fraction. Subsequently, the remaining cell pellet was treated with the appropriate amount of Nuclear Protein Extraction Reagent, vigorously vortexed for 0.5 h at high speed, and then centrifuged at 16,000 g for 10 min at 4 °C to obtain the nuclear protein fraction. The obtained nuclear and cytoplasmic proteins were used for Western blots.

### RNA sequencing

RNA sequencing of human SSCs without or with treatment was performed by BGI (Shenzhen, China). In brief, total RNA was extracted from six samples, and a single-stranded circular DNA library was constructed after passing quality control. Circular DNA molecules were amplified using a rolling circle amplification method to generate DNA nanoballs and followed by sequencing on the BGI self-developed DNBSEQ platform. The raw reads were filtered using SOAPnuke software to obtain high-quality reads. Reference genome alignment was performed using HISAT2 (version: v2.0.4). Ultimately, each sample yielded an average data output of 6.84G. The average alignment rates to the genome and gene set were 83.64% and 69.28%, respectively. DESeq2 was employed for differential gene expression analysis between samples, and clustering analysis was performed using the pheatmap package. Functional enrichment analyses of Gene Ontology (GO) and Kyoto Encyclopedia of Genes and Genomes (KEGG) pathways were conducted using the clusterProfiler package. The results were visualized using R packages, e.g., enrichplot and ggplot2.

### Molecular docking analysis

Molecular docking analysis was conducted using the Home For Researchers online platform (https://www.dockeasy.cn/DockProtein). Initially, we obtained the structural information of USP11 and HOXC5 from the AlphaFold Protein Structure Database (https://alphafold.com/). Subsequently, the acquired structural information was uploaded to the analysis platform, which employed rigid docking to predict complex models of receptors and ligands, to investigate the interaction patterns between USP11 and HOXC5. We used the Z-score as the criterion for selecting the prediction results, where a higher Z-score indicated a better-fitted model.

### Statistical analysis

The statistical analysis of data was conducted with GraphPad Prism 8.0 (CA, USA). The grayscale values of protein blots were quantified using ImageJ (1.58, USA). Each experiment was independently replicated at least three times, and the experimental data were presented as mean ± SD. While differences across many groups were evaluated using analysis of variance (ANOVA), differences between two groups were examined using the t-test. Statistical significance was considered when the *p *value was < 0.05.

### Supplementary Information

Below is the link to the electronic supplementary material.

Supplementary file1 (DOCX 24 KB)

Supplementary file2 (DOCX 6557 KB)

## Data Availability

The data and materials in this article are available with the agreement of corresponding authors.
