# Supplementary material for: USP11 regulates proliferation and apoptosis of human spermatogonial stem cells via HOXC5-mediated canonical WNT/β-catenin signaling pathway
Source: Cell Mol Life Sci. 2024 May 9;81(1):211. doi: 10.1007/s00018-024-05248-6 (PMC11082041; doi:10.1007/s00018-024-05248-6)
Supplement: Supplementary file 1 — Supplementary file1 (DOCX 24 KB) [file 18_2024_5248_MOESM1_ESM.docx]

**Supplemental Tables 1-5**

**Table S1. Sequences of siRNAs targeting *USP11* and *HOXC5***

| **siRNAs** | **Sequences** | |
| --- | --- | --- |
|  | **Sense（5’-3’）** | **Antisense（5’-3’）** |
| USP11 siRNA1 | CCGAUUCUAUUGGCCUAGUAU | AUACUAGGCCAAUAGAAUCGG |
| USP11 siRNA2 | CCGUGAUGAUAUCUUCGUCUA | UAGACGAAGAUAUCAUCACGG |
| USP11 siRNA3 | GCCACAGAAUGAGUCGAAUTT | AUUCGACUCAUUCUGUGGCTT |
| HOXC5 siRNA1 | GCGGAUUGGACUUAAGCAUTT | AUGCUUAAGUCCAAUCCGCTT |
| HOXC5 siRNA2 | GGAUGACCAAACUGCACAUTT | AUGUGCAGUUUGGUCAUCCTT |
| HOXC5 siRNA3 | GCCAACAACUUGUGUCUCATT | UGAGACACAAGUUGUUGGCTT |

**Table S2. Primer sequences of all genes used in this study**

| **Genes** | **Sequences** | |  |
| --- | --- | --- | --- |
|  | **Forward primers (5'-3')** | **Reverse primers (5'-3')** | **Product sizes**  **(bp)** |
| *GPR125* | TGGGTAGGAGTGACAGCTCG | TTGGCCGACTGCCGTAATTC | 190 |
| *UCHL1* | AGCTGAAGGGACAAGAAGTTAG | TTGTCATCTACCCGACATTGG | 265 |
| *MAGEA4* | CTTACCCACTACCATCAGCTTC | TGATGACTCTCTCCAGCATTTC | 212 |
| *SV40* | GAACAGCCCAGCCACTATAA | ACTCCAGCCATCCATTCTTC | 248 |
| *GFRA1* | ACCAAGTACCGCACGCTAAG | TATGGGGAATCCTCCAGCAGA | 233 |
| *THY1* | CGCTAGTGGAGGGTTGGA | TGGAGGCTGTGGGTCAG | 194 |
| *RET* | GCATTGGGCCTCTACTTCT | TTCTCATGCAGCCGTGT | 173 |
| *PLZF* | TTCCTCTCGCCAAAGACC | CTCCAGCATCTTCAGGCA | 150 |
| *DDX4* | GTGGCACAGGTAATGGTGATACTTCTC | TCTCCTCCTTCTGCTTCTGACTTCC | 136 |
| *USP11* | AACGGCGGAACGATTCTGTGATC | CACGGTCCAAGATGCTGCTCAG | 360 |
| *ACTB* | TGGCACCCAGCACAATGAA | CTAAGTCATAGTCCGCCTAGAAGCA | 186 |
| *HOXC5* | AAACTGCACATGAGCCACGAGAC | TTCTTCCACTTCATCCTGCGGTTC | 200 |
| *CCNG1* | CCTTCTGTGTTGGCATTGTCTATC | CAAGCTCTTGCCAGAAGGTCAG | 136 |
| *HOXC8* | AACTCGTCTCCCAGCCTCATGT | TCTAGTTCCAAGGTCTGATACCG | 101 |
| *RBM24* | GTGAACCTGGCATACTTAGGAGC | GCACAAAAGCCTGCGGATAGAC | 139 |
| *SLC12A8* | TGGCGTCTACTCCATGATCTCC | CCGAGATGGATTCAGCAAAGCC | 128 |
| *LRP4* | GTGTGGCAGAACCTTGACAGTC | ACCGCTCTAACTTGGCATTCTCC | 100 |
| *SEPHS1* | GTGTCATTCCTTTGAGGCACGG | AGGTCACTGAGGACATTGGCAC | 121 |
| *SH3RF2* | ATTCCACAGCCGTGGTCAGTCT | CCTTTTGCAGGTCCAGCTCATC | 114 |
| *PKN2* | GCATCACCAACACTAAGTCCACG | GCTTTTGACCGTCCAGGGACAT | 152 |
| *WNT1* | CTCTTCGGCAAGATCGTCAACC | CGATGGAACCTTCTGAGCAGGA | 112 |
| *WNT2* | ATCTGGCTCTGGCTCCCTCTG | CCTGGCACATTATCGCACATCAC | 116 |
| *WNT2B* | TGGATGCCAAGGAGAAGAGGCT | GTACAGGAACCACTCACGCCAT | 133 |
| *WNT3* | GCGTGTTAGTGTCCAGGGAGTT | TGAGGTGCATGTGGTCCAGGAT | 120 |
| *WNT3A* | GGTGGCTGTAGCGAGGACATC | CGTTGTTGTGGCGGTTCATGG | 109 |
| *WNT7A* | AGCGTCTGTTTCTCTCCGTGTG | AATCTCTGCTGTCTGCCTCATACC | 131 |
| *WNT8A* | CATCGAGGAGTGCAAGTTCCAG | GCAGAGCTGATAGCATGTATGAAG | 132 |
| *WNT8B* | AACTGTGGCTGTGATGACTCCC | CGACAAACTGCTTGGAAATCGCC | 112 |
| *WNT10A* | GTGCTCCTGTTCTTCCTACTGC | CCTGGCAATGTTAGGCACACTG | 128 |
| *WNT4* | GCTGGAGAAGTGCGGCTGTGA | CCACAAACGACTGTGAGAAGGC | 116 |
| *WNT6* | GTGCAACTGCACAACAACGAGG | GAAATGGAGGCAGCTTCTGCCA | 127 |
| *WNT11* | ATGTGGCTGCTGACCTCAAGAC | CAGATAGACGAGTTCCGAGTCCTTC | 134 |
| *c-Myc* | GTCAAGAGGCGAACACACAAC | TTGGACGGACAGGATGTATGC | 162 |
| *CCND1* | TCTACACCGACAACTCCATCCG | TCTGGCATTTTGGAGAGGAAGTG | 133 |
| *TCF1* | CTGACCTCTCTGGCTTCTACTC | CAGAACCTAGCATCAAGGATGGG | 192 |
| *AXIN2* | CAAACTTTCGCCAACCGTGGTTG | GGTGCAAAGACATAGCCAGAACC | 156 |
| *MMP9* | GCCACTACTGTGCCTTTGAGTC | CCCTCAGAGAATCGCCAGTACT | 125 |
| *CD44* | CCAGAAGGAACAGTGGTTTGGC | ACTGTCCTCTGGGCTTGGTGTT | 151 |
| *β-catenin* | AAAGCGGCTGTTAGTCACTGG | CGAGTCATTGCATACTGTCCAT | 215 |

**Table S3. The detailed information of the antibodies used in this study for Western bolts**

| **Antibodies** | **Companies** | **Catalog numbers** | **Host/reactivity** | **Dilutions** |
| --- | --- | --- | --- | --- |
| ACTB | CST | 3700S | Mouse | 1:1000 |
| GAPDH | CST | 2118S | Rabbit | 1:1000 |
| Cyclin A2 | CST | 4656T | Mouse | 1:2000 |
| Cyclin B1 | CST | 4138T | Rabbit | 1:1000 |
| Cyclin D1 | CST | 2978T | Rabbit | 1:1000 |
| Cyclin E1 | CST | 4129T | Mouse | 1:1000 |
| Cyclin H | CST | 2927T | Rabbit | 1:1000 |
| CDK2 | CST | 2456T | Rabbit | 1:1000 |
| USP11 | Proteintech | 22340-1-AP | Rabbit | 1:1000 |
| HOXC5 | Santa cruz | sc-517171 | Mouse | 1:500 |
| WNT2B | Bioss | bsm-54266R | Rabbit | 1：1000 |
| WNT3 | Affinity Biosciences | DF13430 | Rabbit | 1:1000 |
| WNT8A | HUABIO | HA721341 | Rabbit | 1:1000 |
| TCF1 | Affinity Biosciences | DF3168 | Rabbit | 1:1000 |
| c-Myc | CST | 13987T | Rabbit | 1:1000 |
| β-catenin | Proteintech | 51067-2-AP | Rabbit | 1:6000 |
| β-tublin | Beyotime Biotechnology | AF2835 | Mouse | 1:1000 |
| H3 | Proteintech | 17168-1-AP | Rabbit | 1:8000 |
| Goat Anti-Mouse HRP | Beyotime Biotechnology | A0126 | Mouse | 1:2000 |
| Goat Anti-Rabbit HRP | Beyotime Biotechnology | A0208 | Rabbit | 1:2000 |

**Table S4. The detailed information of the antibodies used for immunohistochemistry or immunofluorescence**

| **Antibodies** | **Companies** | **Catalog numbers** | **Host/reactivity** | **Dilutions** |
| --- | --- | --- | --- | --- |
| THY1 | LifeSpan BioSciences | LS-C45431 | Mouse | 1:50 |
| UCHL1 | Bio-rad | MCA4750GA | Mouse | 1:200 |
| VASA | Santa | SC26877 | Mouse | 1:50 |
| PLZF | abcam | ab104854 | Mouse | 1:50 |
| GPR125 | abcam | ab51705 | Rabbit | 1:50 |
| GFRA1 | Santa cruz | sc-10716 | Rabbit | 1:50 |
| PCNA | abcam | ab29 | Mouse | 1:50 |
| SV40 | Santa cruz | sc-147 | Mouse | 1:50 |
| USP11 | Proteintech | 22340-1-AP | Rabbit | 1:100 |
| HOXC5 | bioss | bs-11589R | Rabbit | 1:50 |
| HOXC5 | Santa cruz | sc-517171 | Mouse | 1:20 |
| β-catenin | Proteintech | 51067-2AP | Rabbit | 1:100 |
| AlexaFluor488 | Thermo scientific | A21202 | Mouse | 1:1000 |
| AlexaFluor488 | Thermo scientific | A21206 | Rabbit | 1:1000 |
| AlexaFluor555 | Thermo scientific | A31572 | Rabbit | 1:1000 |
| AlexaFluor555 | Thermo scientific | A31570 | Mouse | 1:1000 |

**Table S5. Transcription levels of *HOX* family genes in RNAseq data**

| **Gene IDs** | **Gene Symbols** | **log2**  **(siRNA treat/control siRNA)** | **Control**  **Average TPM** | **Treat Average TPM** | ***p* value**  **(siRNA treat/control siRNA)** |
| --- | --- | --- | --- | --- | --- |
| 3222 | *HOXC5* | -1.30731 | 2.866667 | 1.093333 | 0.004921 |
| 3224 | *HOXC8* | -1.27563 | 8.633333 | 3.363333 | 6.17E-10 |
| 3225 | *HOXC9* | -0.73378 | 3.626667 | 2.06 | 0.048299 |
| 3232 | *HOXD3* | -0.47828 | 9.59 | 6.37 | 0.003013 |
| 3221 | *HOXC4* | -0.26009 | 9.34 | 7.446667 | 0.181886 |
| 3235 | *HOXD9* | -0.23025 | 23.25667 | 18.68667 | 0.057342 |
| 3233 | *HOXD4* | 0.288137 | 48.37333 | 56.96 | 2.07E-06 |
| 3234 | *HOXD8* | 0.300082 | 27.62 | 32.04333 | 0.001979 |
