# Supplementary material for: USP11 regulates proliferation and apoptosis of human spermatogonial stem cells via HOXC5-mediated canonical WNT/β-catenin signaling pathway
Source: Cell Mol Life Sci. 2024 May 9;81(1):211. doi: 10.1007/s00018-024-05248-6 (PMC11082041; doi:10.1007/s00018-024-05248-6)
Supplement: Supplementary file 2 — Supplementary file2 (DOCX 6557 KB) [file 18_2024_5248_MOESM2_ESM.docx]

**USP11 regulates proliferation and apoptosis of human spermatogonial stem cells via HOXC5-mediated canonical WNT/β-catenin signaling pathway**

Jun Gao, Zhipeng Xu, Weijie Song, Jiwei Huang, Wei Liu, Zuping He^#^ and Leye He^#^

**Supplemental Figures 1-8 and Figure legends**

**Supplemental Figures 1-8**

**Fig. S1**

**
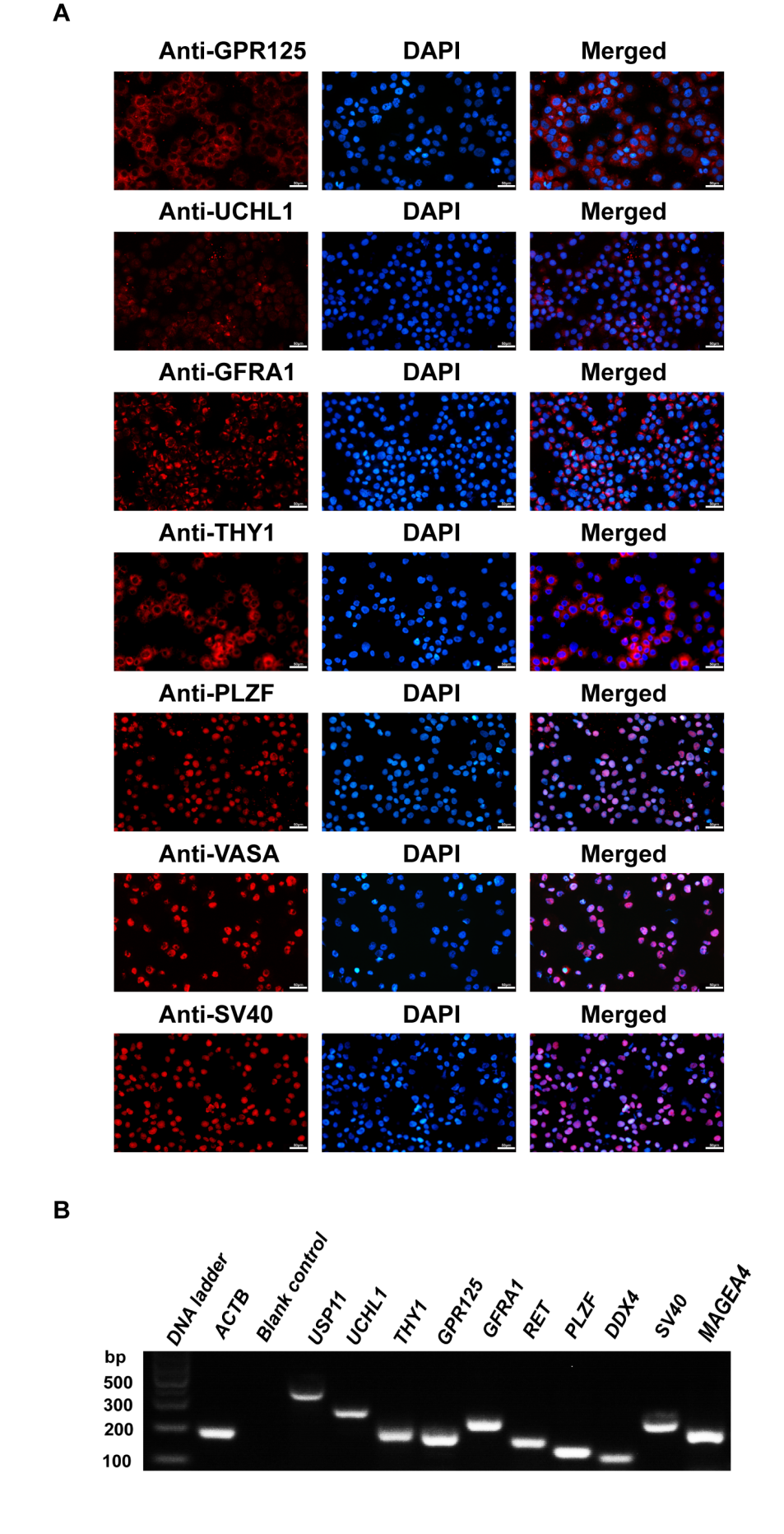
**

**Fig. S1.** **Identification of human SSC line**. **A.** Immunofluorescence illustrated the expression of proteins, including GFR125, UCHL1, GFRA1, THY1, PLZF, VASA, and SV40, in human SSC line. The red color represented the positive cells for the proteins. The cell nuclei were stained with DAPI. Scale bars: 50 µm. **B.** RT-PCR showed the transcripts of genes for human SSCs in human SSC line. ACTB was used as a loading control of total RNA.

**Fig. S2**


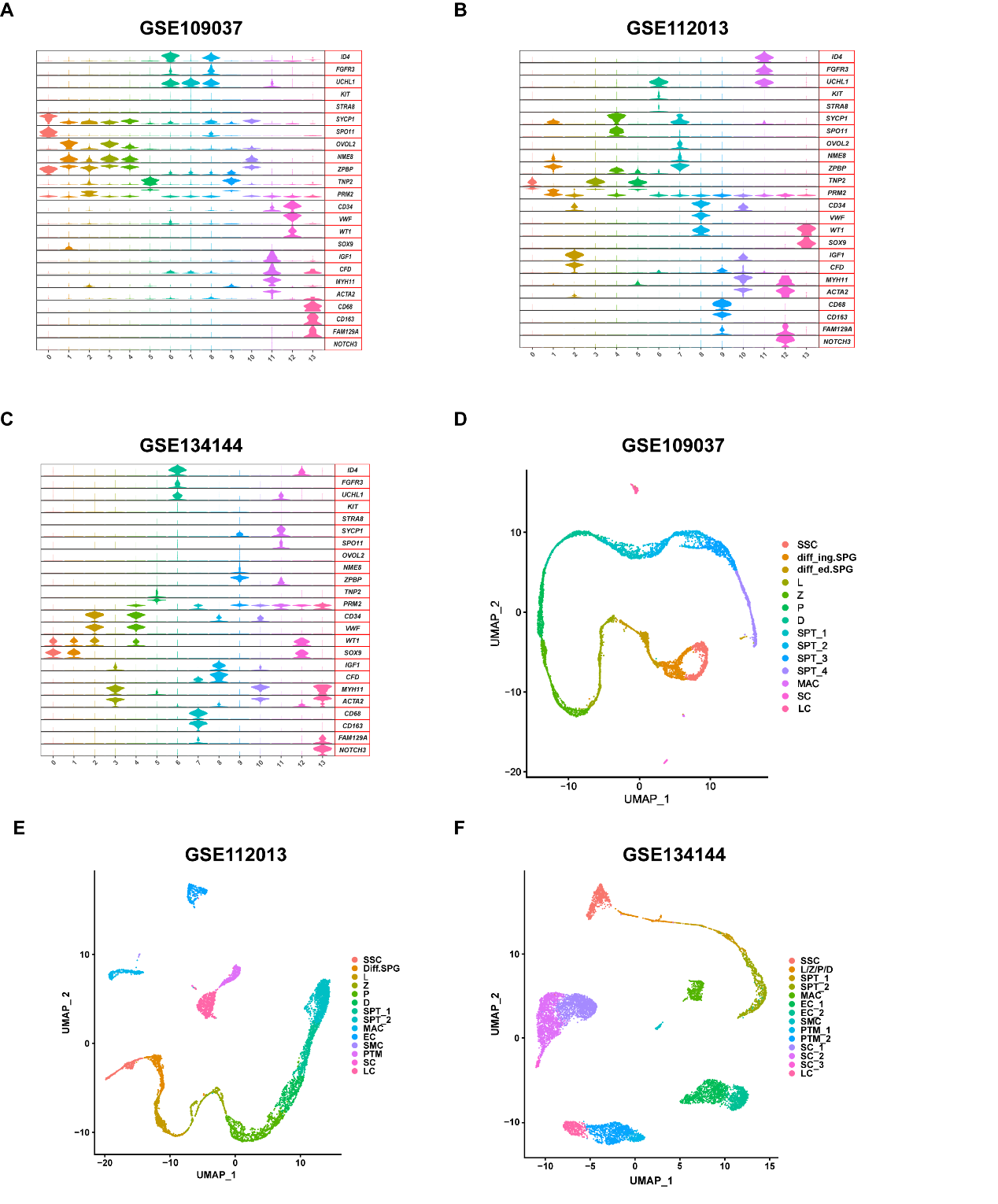


**Fig.S2.** **The comprehensive analysis of three human testicular single-cell transcriptomic datasets included GSE109037, GSE112013, and GSE134144**. **A-C.** The distribution of marker genes across all testicular cells was visualized through violin plots. **D-F.** UMAP clustering and visualization analysis of human testicular single-cell sequencing data. We categorized all testicular cells into 14 cellular clusters, with cells in the same cluster represented by the same color, and each data point indicated an individual cell. Notes: SSC: spermatogonial stem cells; Diff_ing.SPG, differentiating spermatogonia; Diff_ed.SPG, differentiated spermatogonia; L, leptotene spermatocytes; Z, zygotene spermatocytes; P, pachytene spermatocytes; D, diplotene spermatocytes; SPT, spermatids; MAC; mast cells; SC, Sertoli cells; LC, Leydig cells; PTM, peritubular myoid cells; EC, endothelial cells; SMC, smooth muscle cells; MAC, macrophages.

**Fig. S3**

**
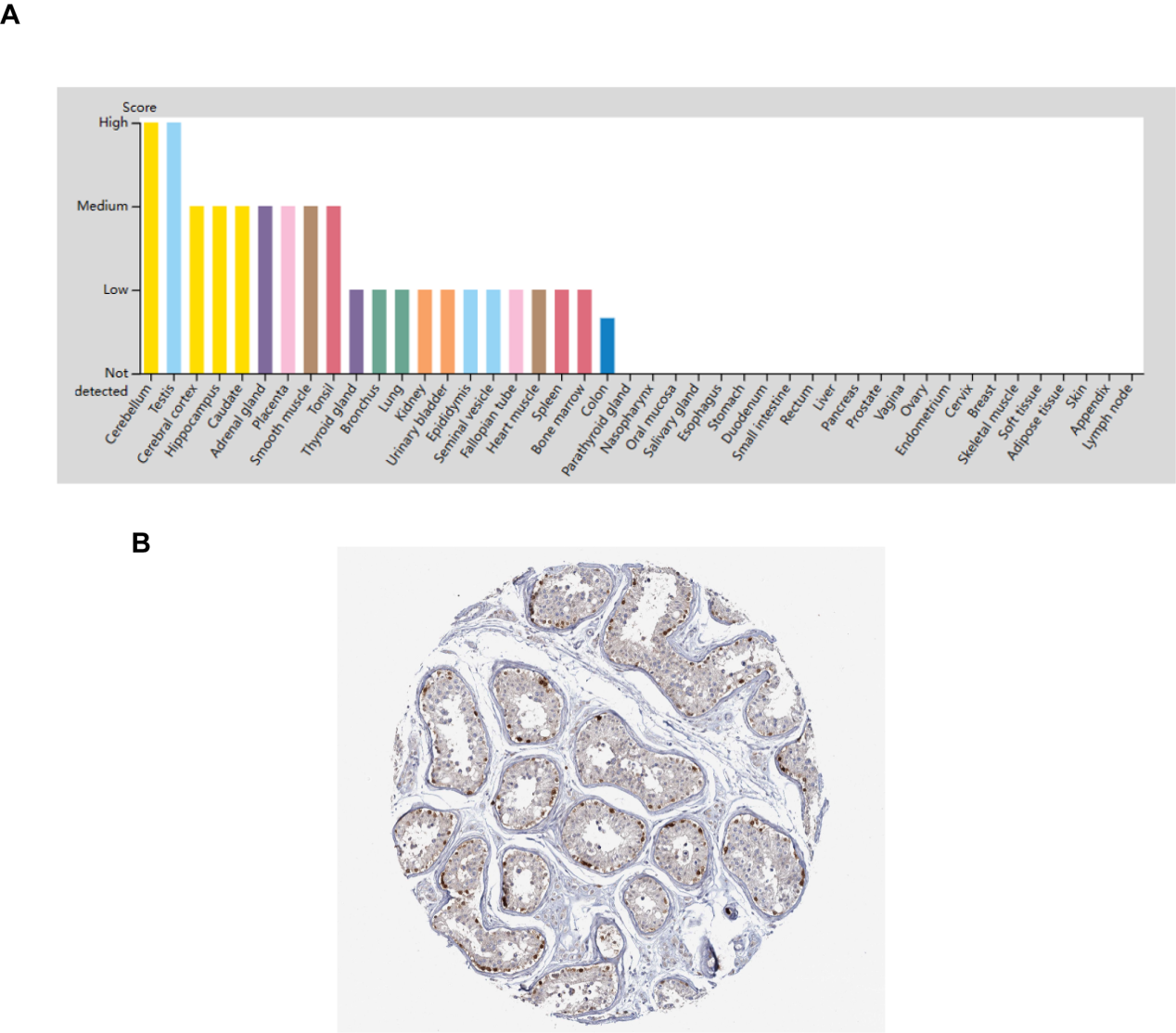
**

**Fig.S3. The expression profile of USP11 in the HPA database**. **A.** Expression of USP11 in numerous tissues from the HPA database. **B.** Immunohistochemistry revealed the expression of USP11 in spermatogonia located along the basement membrance of seminiferous tubules according to the HPA database.

**Fig. S4**


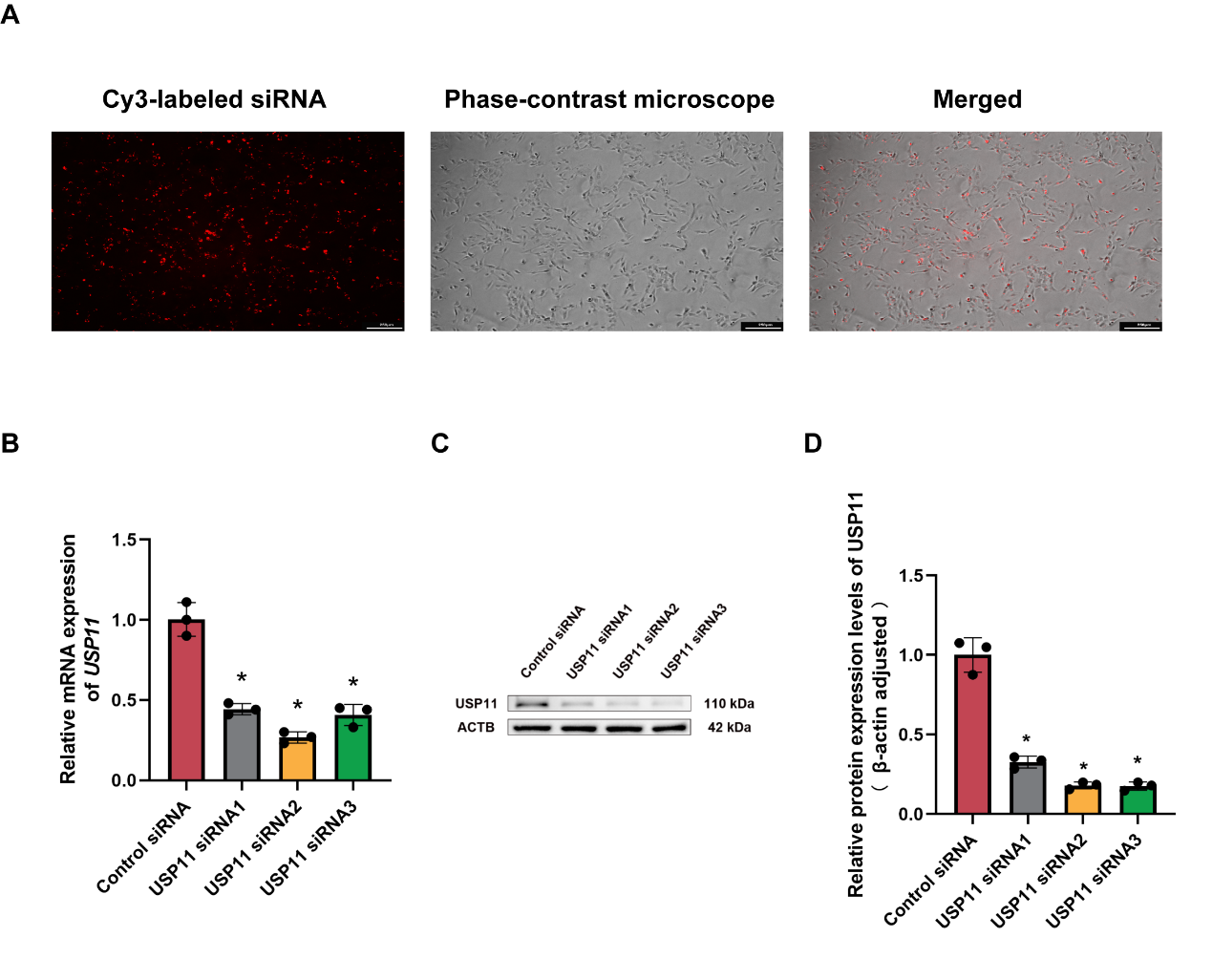


**Fig.S4. Silencing efficiency of USP11 siRNAs in human SSCs**. **A.** Validation of the transfection system’s effectiveness in human SSCs using Cy3-labeled siRNAs. The red signal indicated successful siRNA transfection. Scale bars: 250 µm. **B.** Detection of the altered transcript levels of *USP11* in human SSCs after USP11 siRNA1-3 transfection by RT-qPCR. **C** and **D.** The protein expression of USP11 in human SSCs after transfection with USP11 siRNA1-3 was measured by Western blots. “*” denoted a statistically significant difference with a *p*-value < 0.05.

**Fig. S5**


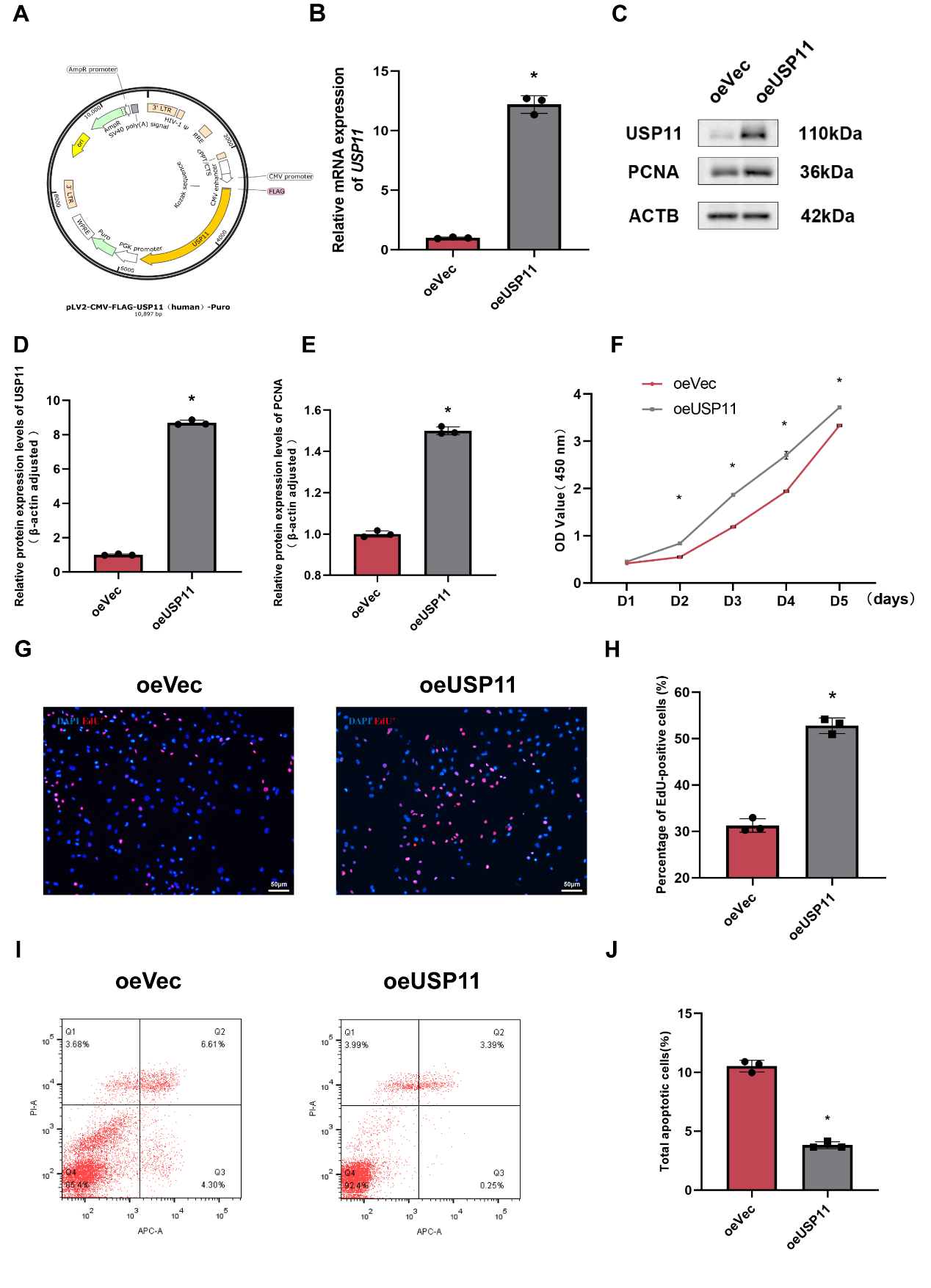


**Fig.S5. The impact of USP11 overexpression on proliferation and apoptosis of human SSCs**. **A.** Plasmid map for USP11 overexpression. **B-D.** Validation of USP11 overexpression efficiency in human SSCs at transcriptional and protein levels. **C**, **E.** Impact of USP11 overexpression on PCNA protein expression in human SSCs. **F.** Cell proliferation curve after overexpression of USP11 in human SSCs. **G** and **H.** Effect of USP11 overexpression on DNA synthesis in human SSCs. The red signal represented EDU-positive staining. Scale bars: 50 µm. **I** and **J.** Assessment of apoptosis in human SSCs overexpressing USP11. “*” denoted a statistically significant difference with a *p*-value < 0.05.

**Fig. S6**


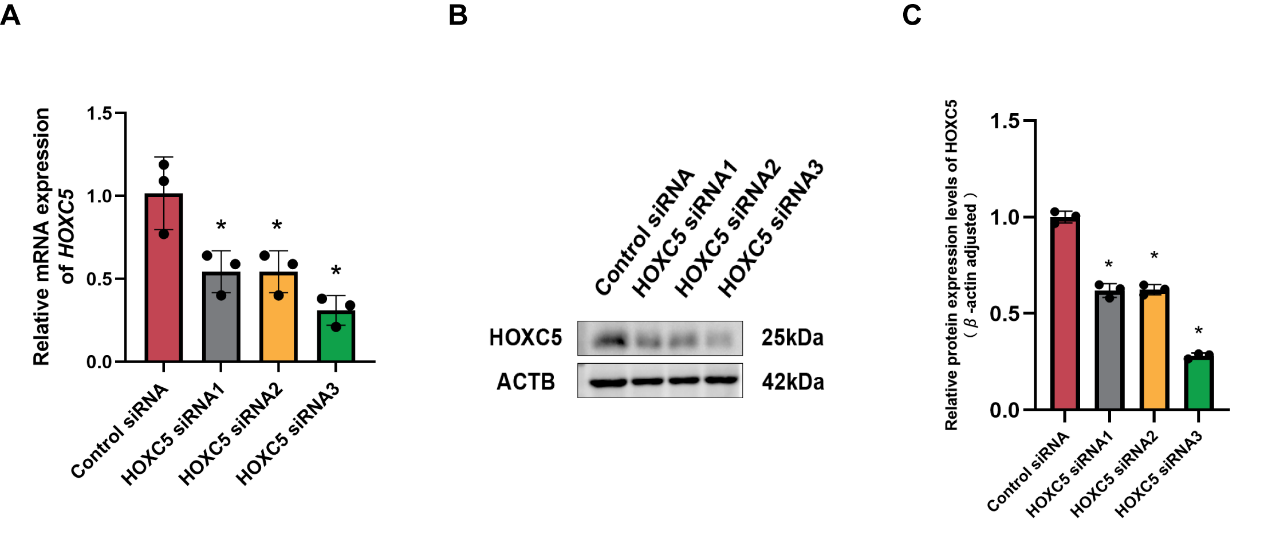


**Fig.S6. HOXC5 siRNA1-3 knockdown efficiency in human SSCs**. **A.** Transcriptional changes of *HOXC5* in human SSCs following HOXC5 siRNAs were detected using RT-qPCR. **B** and **C.** The protein expression of HOXC5 in human SSCs by HOXC5 siRNAs was assessed by Western blots. “*” denoted a statistically significant difference with a *p*-value < 0.05.

**Fig. S7**


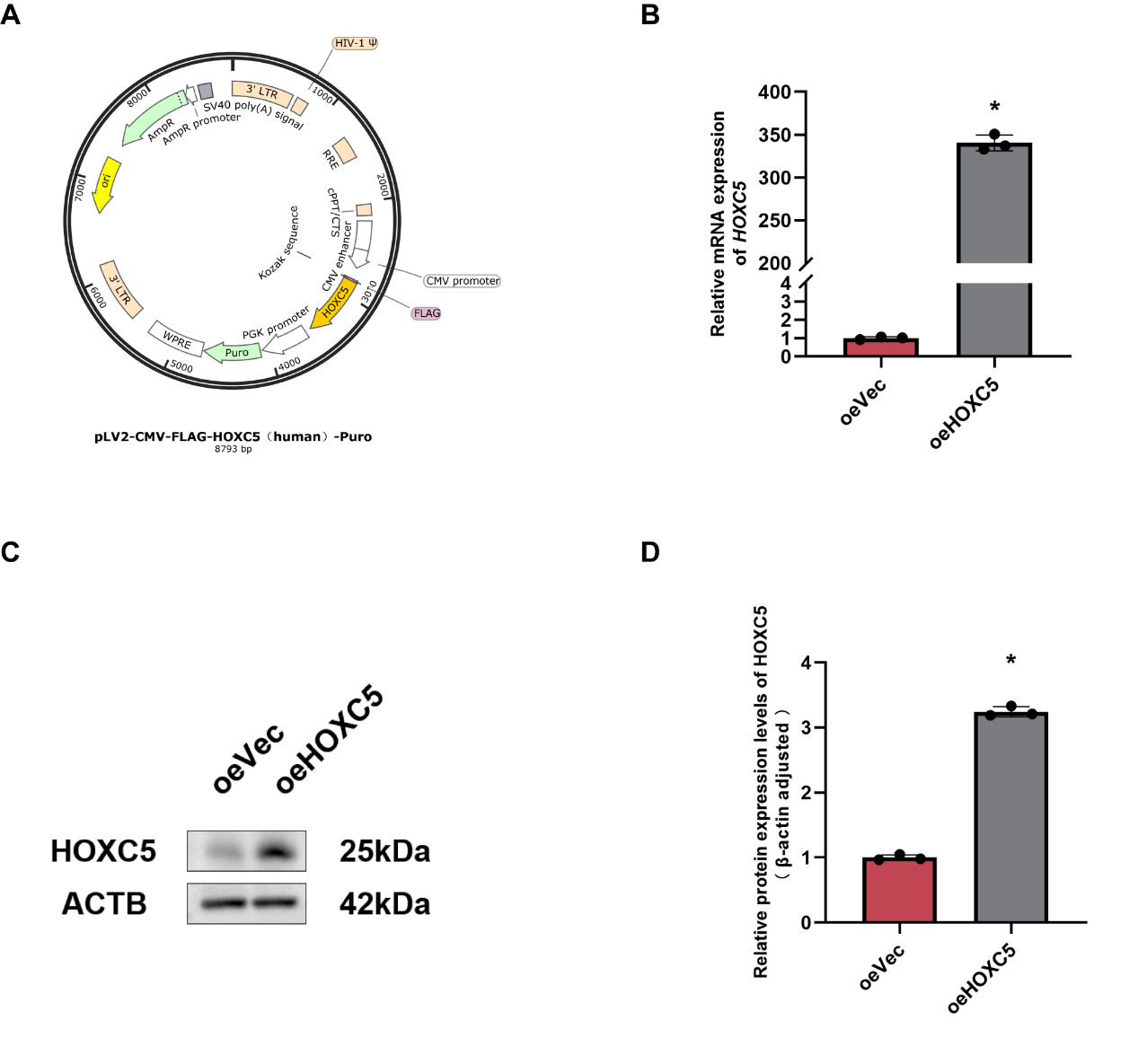


**Fig. S7.** Overexpression efficiency of HOXC5 in human SSCs. **A.** Sequence map of the HOXC5 overexpression plasmid. **B.** Changes in *HOXC5* mRNA levels in human SSCs after overexpression as detected by qPCR. **C** and **D.** Western blots assessed protein expression of HOXC5 in human SSCs following HOXC5 overexpression. “*” indicated a statistically significant difference with a *p*-value < 0.05.

**Fig. S8**


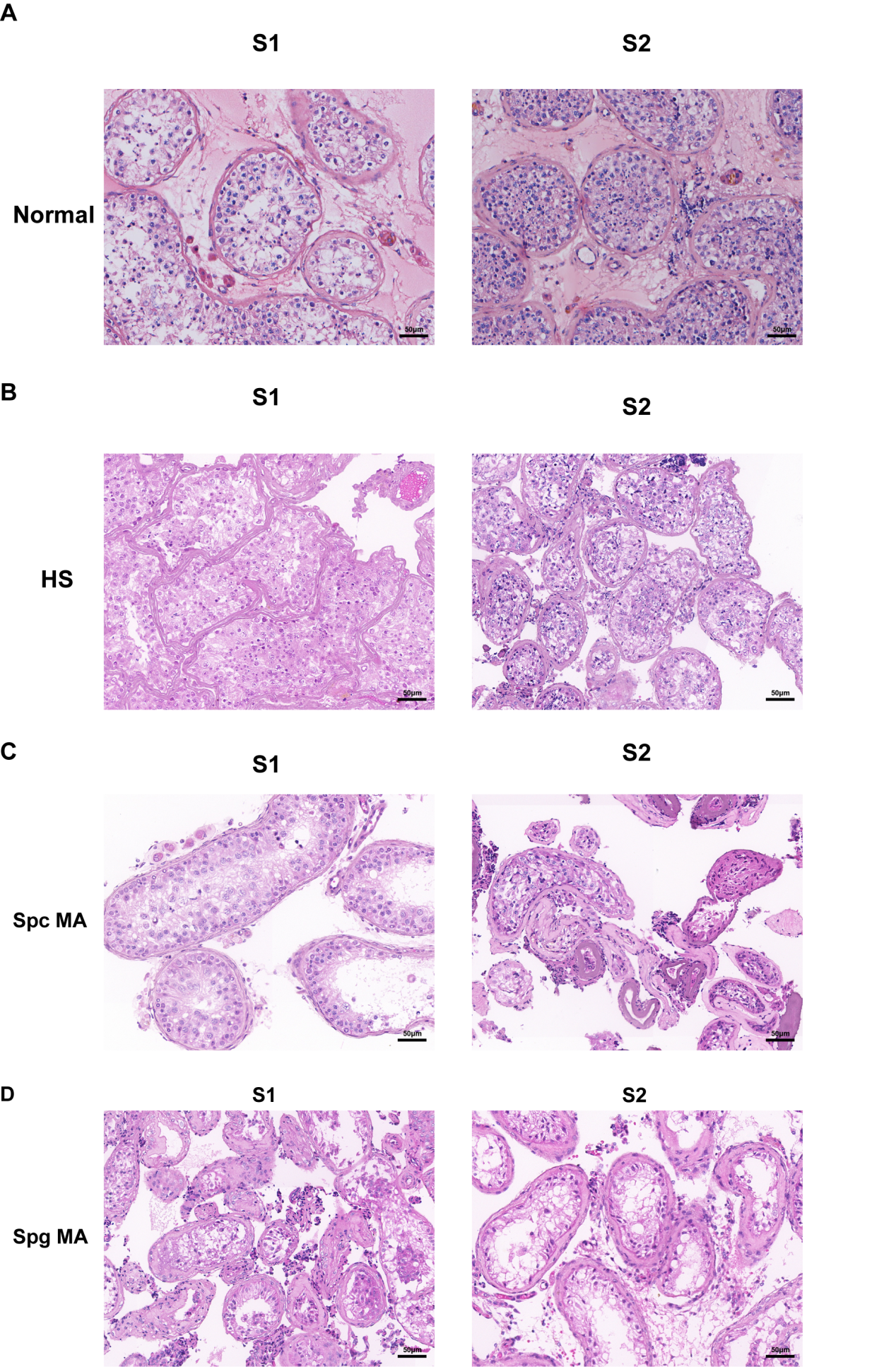


**Fig.S8. Representative morphological features of testicular tissues from NOA patients with varying spermatogenic disorders**. **A.** Typical hematoxylin-eosin (H&E) staining of testicular tissues with normal spermatogenic function. **B.** Representive H&E staining of testicular tissue with hypospermatogenesis (HS). **C.** Typical H&E staining of testicular tissues with spermatogenic arrest at the spermatocyte maturation stage (Spc MA). **D.** Typical H&E staining of testicular tissue with spermatogenic arrest at the spermatogonial maturation stage (Spg MA). Notes: S1 represented sample 1, and S2 denoted sample 2. Scale bars: 50 µm
